# Author Correction: Characterization of an RNA binding protein interactome reveals a context-specific post-transcriptional landscape of *MYC*-amplified medulloblastoma

**DOI:** 10.1038/s41467-023-35816-6

**Published:** 2023-01-10

**Authors:** Michelle M. Kameda-Smith, Helen Zhu, En-Ching Luo, Yujin Suk, Agata Xella, Brian Yee, Chirayu Chokshi, Sansi Xing, Frederick Tan, Raymond G. Fox, Ashley A. Adile, David Bakhshinyan, Kevin Brown, William D. Gwynne, Minomi Subapanditha, Petar Miletic, Daniel Picard, Ian Burns, Jason Moffat, Kamil Paruch, Adam Fleming, Kristin Hope, John P. Provias, Marc Remke, Yu Lu, Tannishtha Reya, Chitra Venugopal, Jüri Reimand, Robert J. Wechsler-Reya, Gene W. Yeo, Sheila K. Singh

**Affiliations:** 1 Centre for Discovery in Cancer Research (CDCR), McMaster University, Hamilton, ON Canada; 2 Department of Biochemistry and Biomedical Sciences, McMaster University, Hamilton, ON Canada; 3 Surgery, Faculty of Health Sciences, McMaster University, Hamilton, ON Canada; 4 Computational Biology Program, Ontario Institute for Cancer Research, Toronto, ON Canada; 5 Department of Medical Biophysics, University of Toronto, Toronto, ON Canada; 6 University Health Network, Toronto, ON Canada; 7 Vector Institute Toronto, Toronto, ON Canada; 8 Department of Cellular and Molecular Medicine, University of California at San Diego, La Jolla, CA USA; 9 Stem Cell Program, University of California San Diego, La Jolla, CA USA; 10 Sanford Consortium for Regenerative Medicine, La Jolla, CA USA; 11 Michael G DeGroote School of Medicine, McMaster University, Hamilton, ON Canada; 12 Tumor Initiation and Maintenance Program, National Cancer Institute-Designated Cancer Center, Sanford Burnham Prebys Medical Discovery Institute, La Jolla, CA USA; 13 Departments of Pharmacology and Medicine, University of California San Diego School of Medicine, Sanford Consortium for Regenerative Medicine, La Jolla, CA USA; 14 Donnelly Centre, Department of Molecular Genetics, University of Toronto, Toronto, ON Canada; 15 Department of Pediatric Oncology, Hematology, and Clinical Immunology, Medical Faculty, University Hospital Düsseldorf, Düsseldorf, Germany; 16 Department of Chemistry, CZ Openscreen, Faculty of Science, Masaryk University, Kamenice 5, 625 00 Brno, Czech Republic; 17 International Clinical Research Center, St. Anne’s University Hospital in Brno, 602 00 Brno, Czech Republic; 18 Departments of Pediatrics, Hematology and Oncology Division, McMaster University, Hamilton, ON Canada; 19 Department of Neuropathology, McMaster University, Hamilton, ON Canada; 20 Department of Molecular Genetics, University of Toronto, Toronto, ON Canada; 21 Department of Pediatrics, McMaster University, Hamilton, ON Canada; 22 Present Address: Herbert Irving Comprehensive Cancer Center, Department of Physiology and Cellular Biophysics, Columbia University Medical Center, New York, NY USA

**Keywords:** Paediatric cancer, Neural stem cells, Cancer stem cells

Correction to: *Nature Communications* 10.1038/s41467-022-35118-3, published online 06 December 2022

In this article, the affiliations ‘Computational Biology Program, Ontario Institute for Cancer Research, Toronto, ON, Canada’ and ‘Department of Medical Biophysics, University of Toronto, Toronto, ON, Canada’ for Helen Zhu were missing.

In this article, the affiliation ‘Department of Molecular Genetics, University of Toronto, Toronto, ON, Canada’ for Jüri Reimand was missing.

The original article has been corrected.

