# Supplementary material for: Characterization of an RNA binding protein interactome reveals a context-specific post-transcriptional landscape of MYC-amplified medulloblastoma
Source: Nat Commun. 2022 Dec 6;13:7506. doi: 10.1038/s41467-022-35118-3 (PMC9726987; doi:10.1038/s41467-022-35118-3)
Supplement: Supplementary file 1 — Supplementary Information [file 41467_2022_35118_MOESM1_ESM.pdf]

Characterization of an RNA binding protein interactome reveals a context-specific post-transcriptional landscape of MYC-amplified medulloblastoma

Kameda-Smith et al.

Supplementary Information

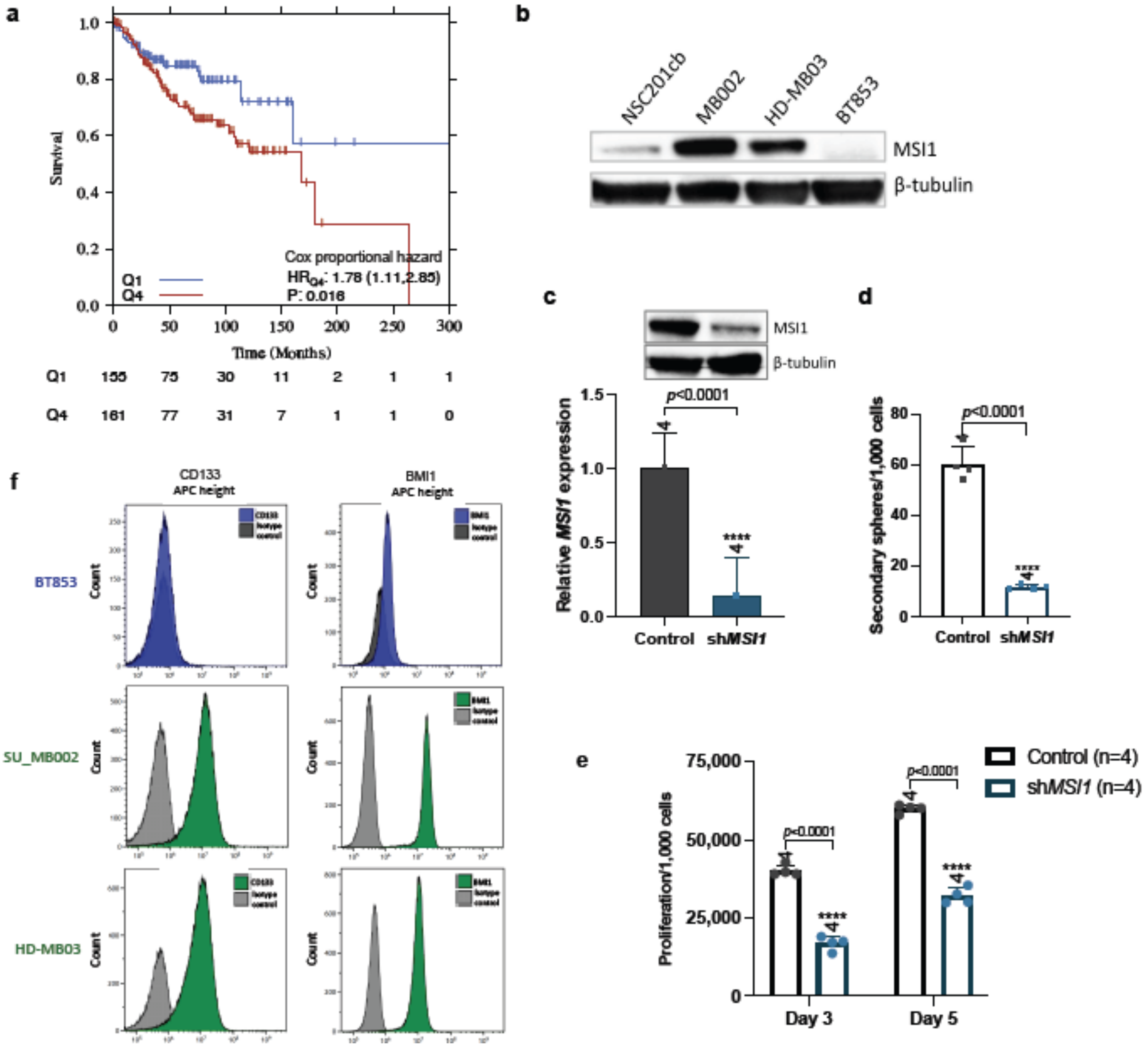

Supplementary Figure 1: *MSI1* is highly expressed in NSC and G3 MB. **a.** Survival data from the Cavalli transcriptomic data showing a significant increase in survival in the top quartile vs bottom quartile of MSI1 expression (HR: 1.78, *p*=0.016), **b**. Western immunoblot of MB cell lines demonstrating high protein expression of MSI1 in G3 MB lines (SU_MB002, HD-MB03) *vs* a cerebellar neural stem line (NSC201cb) and non-G3 MB line (BT853), **c-e.** sh*MSI*1KD of human NSC results in significant reduction in transcript and protein expression with abrogation of stem cell properties: secondary sphere formation (*p*<0.0001) and proliferation day 5 post-transduction (*p*<0.0001), **f.** Flow cytometric analysis demonstrating higher CD133 and BMI1 in G3 MB (SU_MB002 and HD-MB03) as compared to non-G3 MB (BT853) samples.

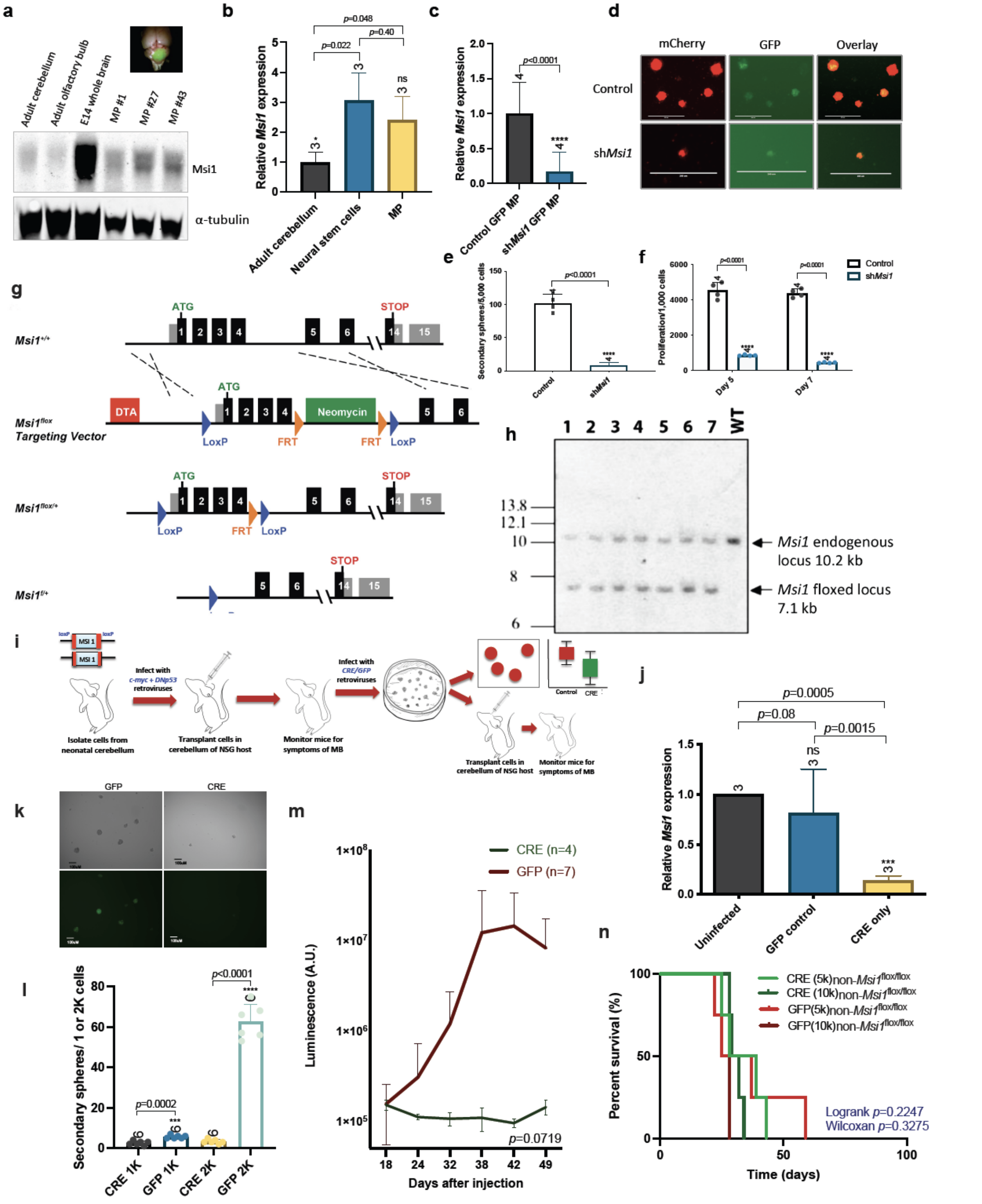

Supplementary Figure 2: Cre mediated *Msi1*$^{fl/fl}$ KO in MP cells impair the key stem cell property of self- renewal and abrogates the MP's capacity for tumor formation. a. Western immunoblot showing greater Msi1 protein abundance in MP tumors as compared to adult mouse cerebellum and olfactory bulb but not embryonic whole brain, **b.** RT-qPCR showing a comparison of the relative expression of Msi1 in

adult mouse cerebellum and neural stem cells (p=0.022) as compared to MP cells (p=0.40), **c-f.** Significant reduction in MSI1 transcript expression after shMSI1 inhibition of MP cells (mCherry fluorescent protein chromophore used to identify cells with both myc amplification and p53 mutation) resulting in reduction in stem cell properties of secondary sphere formation (*p*<0.0001) and proliferation (*p*<0.0001), **g.** Schematic of targeting strategy for the generation of *Msi1* conditional knockout mice (*Msi1*$^{flox/flox}$), **h.** Southern blot analysis of heterozygous mice carrying the floxed allele after genomic DNA digestion, **i.** Schematic of MP model adapted from Pei *et al*, 2012[1], **j.** RT-qPCR *Msi1*$^{flox/flox}$ KO validation of *Msi1*$^{flox/flox}$ KO *vs* control showing Cre-mediated excision lowers *Msi1* mRNA levels compared to control GFP cells (*p*=0.0015), **k.** Primary neurosphere formation after *Msi1*$^{flox/flox}$ KO of MP tumor cells showing greater neurosphere forming capacity of control GFP *Msi1* MP than Cre-mediated *Msi1* KO MP cells, **l.** Quantification of primary sphere formation between CRE and GFP infected MP cells (*p*<0.0001), **m.** Luminescence graph of Cre *vs* GFP mice showing reduced Luciferin signal in the GFP control mice compared to the Cre-Msi1-excised mice (Paired t-test, *p*=0.0719), **n.** Kaplan-Meier survival plot showing no survival benefit in Cre-treated cells and therefore Cre toxicity does not explain survival benefit (*p*=0.2247).

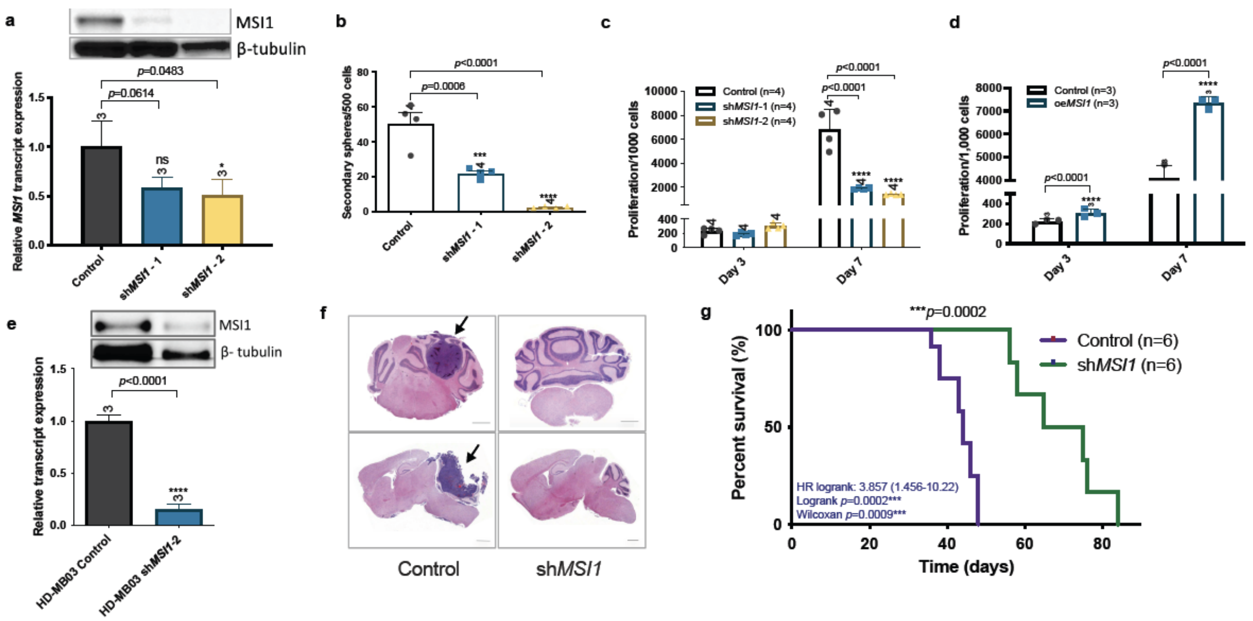

Supplementary Figure 3: shMSI1 KD in G3 MB BTIC impair the key stem cell property of self-renewal and abrogates capacity for tumor formation. **a-c**. sh*MSI*1KD of SU_MB002 results in significant reduction in transcript and protein expression with similar abrogation of stem cell properties: secondary sphere formation in sh*MSI1*-2 (*p*<0.0001) and proliferation at day 7 post-transduction (*p*<0.0001), **d.** Over-expression of *MSI1* in an already overexpressed system pushes the cell to further increase proliferation capacity (*p*<0.0001), **e.** *In vivo* experimentation repeated in a second G3 MB patient derived cell line, HD-MB03 showing significant *MSI1* KD at both the transcript and protein levels after sh*MSI1* inhibition (*p*<0.0001), and **f.** large tumors in the control arms as compared to the sh*MSI1* brains after orthotopic transplantation of 1000 cells per mouse (bars represent 100µm), **g.** HD-MB03 sh*MSI1* KD engrafted mice conferring a significant survival benefit *(p*=0.0002).

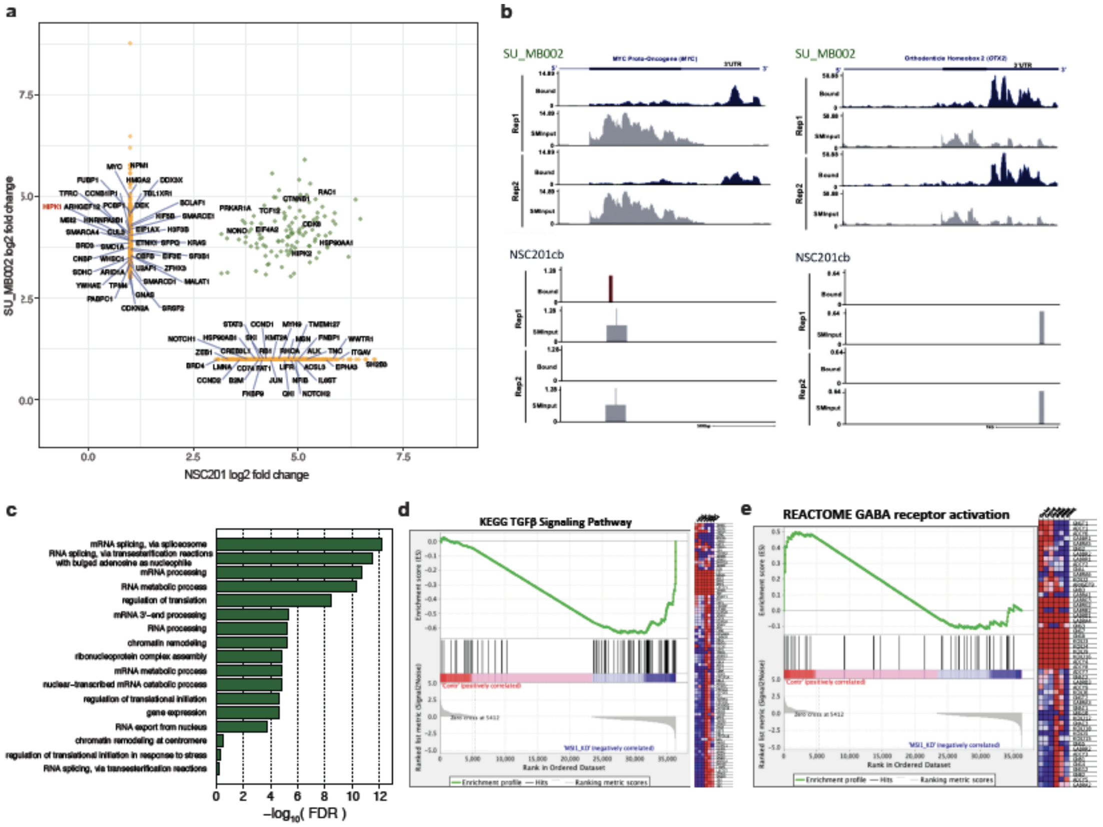

Supplementary Figure 4: eCLIP analysis reveals differentially bound transcripts in NSC and G3 MB with key MSI1 bound transcripts involved in TGF-β pathway, GABA activation. **a.** Analysis of the eCLIP binding targets identifying key cancer and stem cell associated genes that are unique and shared by both SU_MB002 and NSC201cb, **b.** eCLIP biological replicates of NSC201 and SU_MB002 showing CLIPper-identified MYC and OTX3 clusters indicating differentially enriched 3' UTR binding sites between NSC and G3 MB, **c.** EnrichR analysis identifying dysregulation of RNA processes within the eCLIP bound differentially expressed genes in RNA-seq (FC>1.5, *p*<0.05), **d-e.** GSEA showing dysregulation of genes known to be associated with G3 MB, the TGF-β and GABA activation pathways after sh*MSI1* inhibition

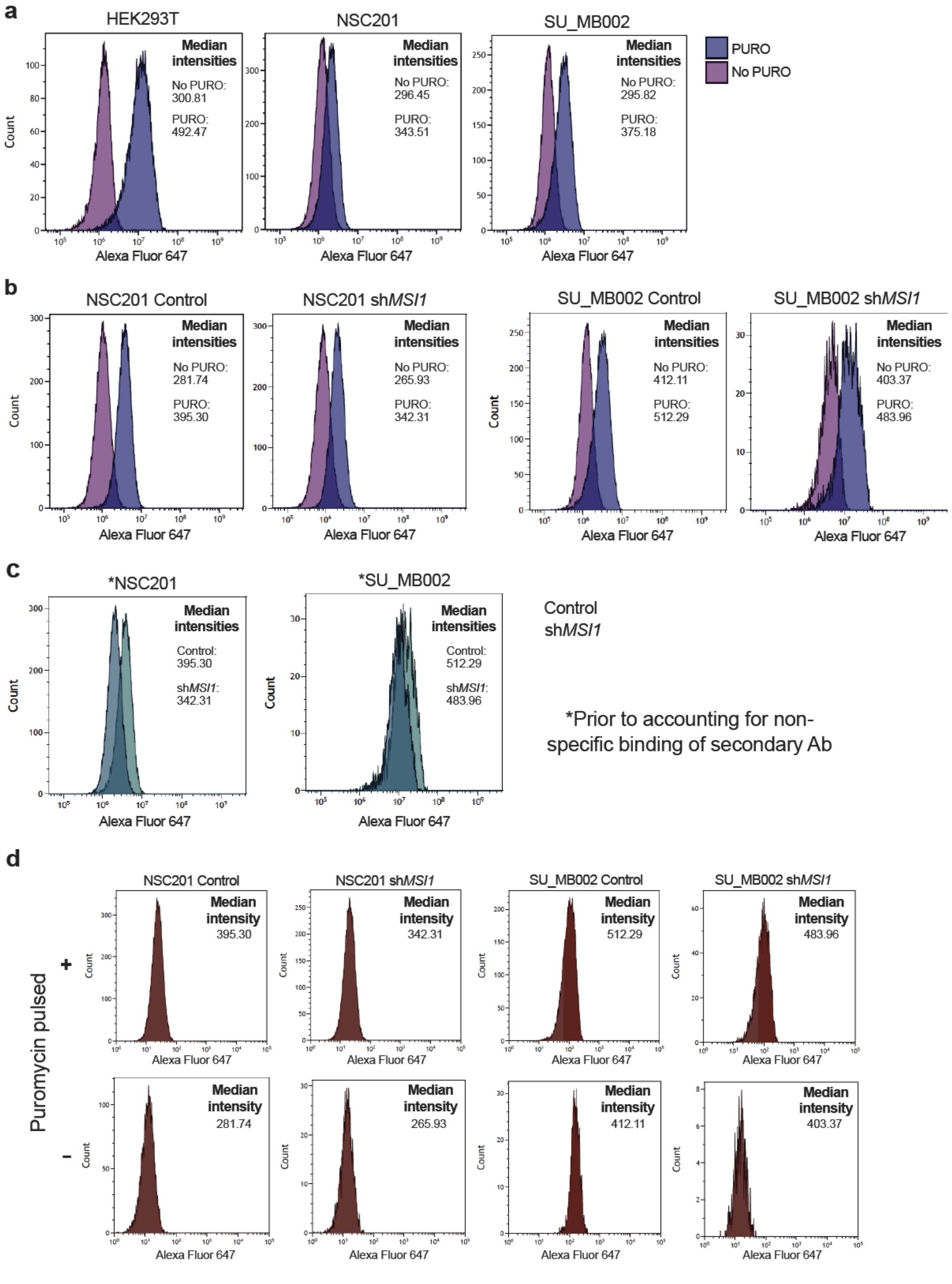

Supplementary Figure 5: SuNSET FACs assay suggests an increase in nascent polypeptide production in sh*MSI1* inhibited neural systems. **a.** Histogram of HEK validation of puromycylation with comparisons with untransduced NSC and SU_MB002, **b.** NSC201 and SU_MB002 control *vs* sh*MSI1* transduced cells showing rightward shift in puromycin pulsed samples, **c.** Composite histograms of NSC andsU_MB002 control *vs* sh*MSI1* puromycin pulsed samples showing a slight leftward shift in the sh*MSI1* samples however, **d.** Histograms of cells that were stained only with the secondary antibody showing slight increase in non-specific staining of all 4 control samples requiring normalization for Figure 4D.

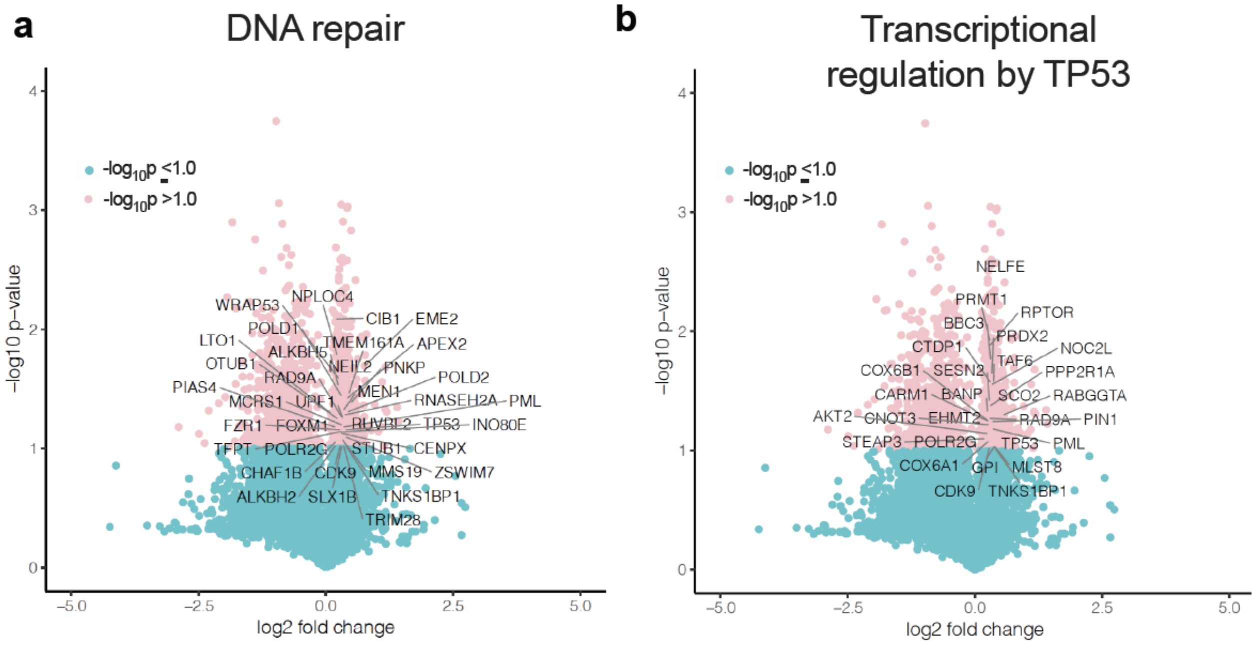

Supplementary Figure 6: Pathways analysis of upregulated transcripts associated with a polysome fraction identifies genes involved life-sustaining processes. a. Upregulated genes associated with DNA repair, **b.** Upregulated genes associated with transcriptional regulation by TP53.

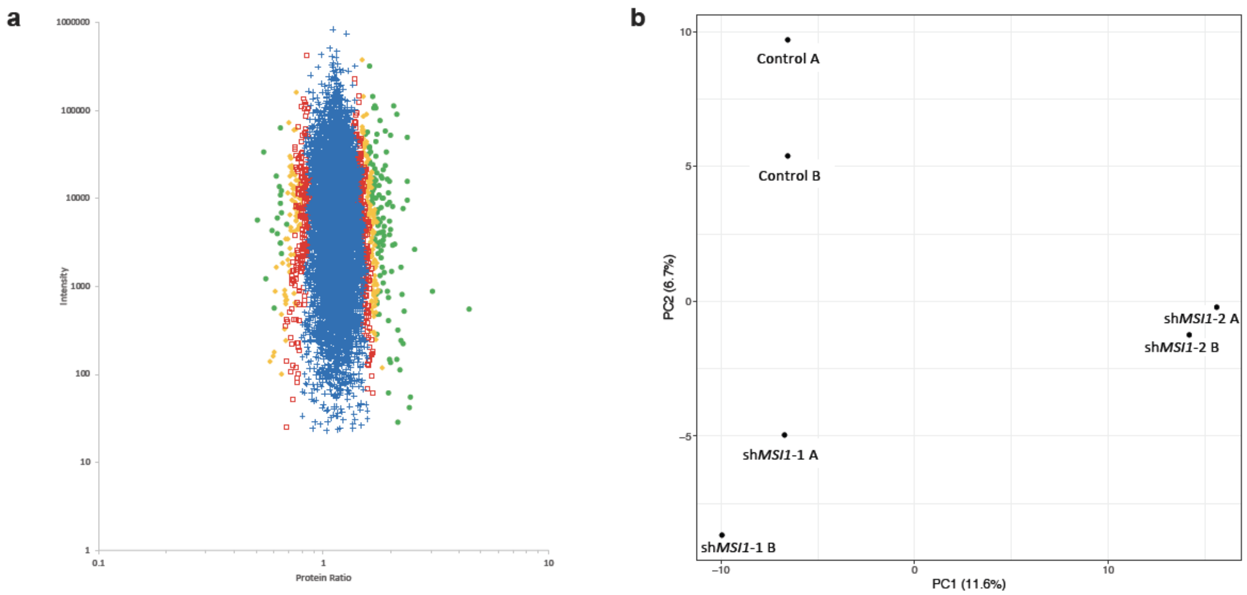

Supplementary Figure 7: MaxQUANT and PERSEUS analysis of proteomic data suggest an increase in steady state protein after sh*MSI1* inhibition. a. MaxQUANT and PERSEUS analyzed control and sh*MSI1* samples showing extensive changes in the proteome after sh*MSI1* inhibition with a rightward shift of protein abundance. These are normalized protein ratios plotted against summed peptide intensities and the data points are colored by their 'significance B' value (blue crosses: >0.05, red squares between 0.05 and 0.01, yellow diamonds between 0.01 and 0.001 and green circles <0.01), **b.** Principal component analysis (PCA) analysis illustrating cells with greatest MSI1 KD separates further from control samples.

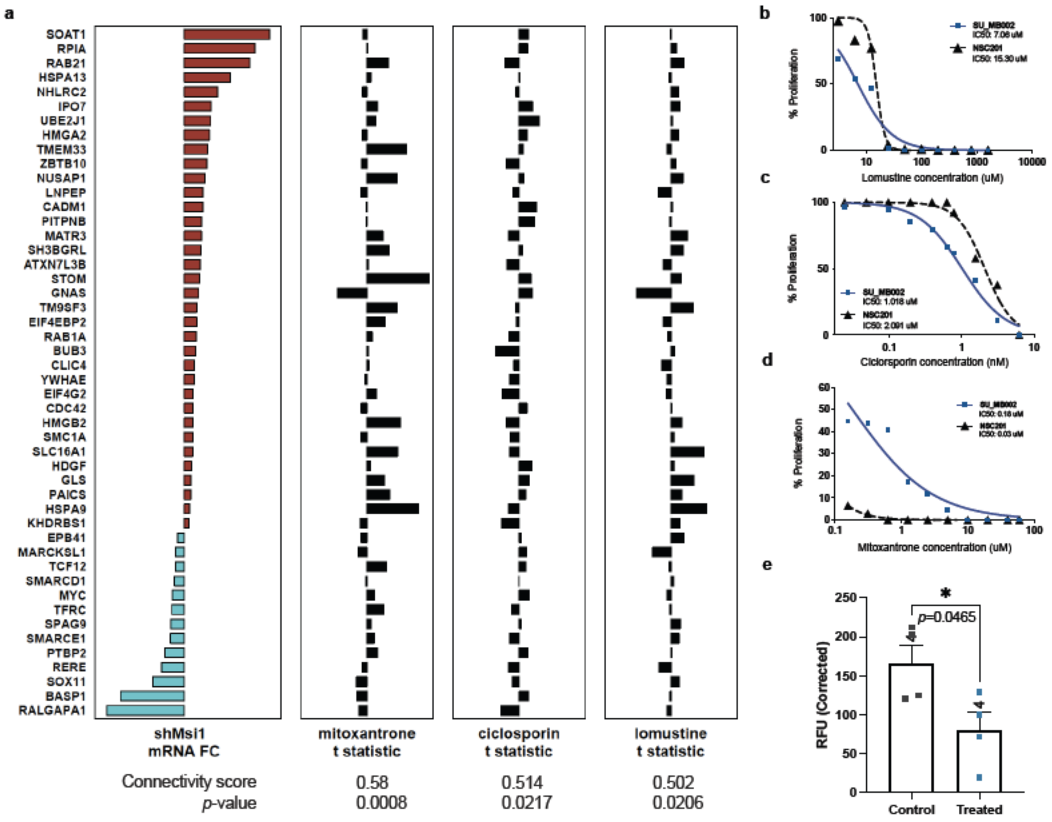

Supplementary Figure 8: CMAP analysis repurposing drugs to target MSI1 shows a therapeutic window between G3 MB and NSC to be too narrow. a. CMAP analysis identifying top differentially expressed transcripts after sh*MSI1* inhibition (Connectivity score and *p*-value as labelled for repurposed drugs known to target neural origin tumors – mitoxantrone, ciclosporin and lomustine), **b-f.** Differential IC50 analyses of mitoxantrone, ciclosporin and lomustine in SU_MB002 *vs* human NSC showing MSI1 targeting is not a viable strategy for drug discovery due to the vulnerability of resident human NSC to the repurposed drugs with similar changes in transcriptomic profile after MSI1 inhibition, **e**. Corrected RFU of NSC201 after treatment with mitoxantrone (*p*=0.0465).

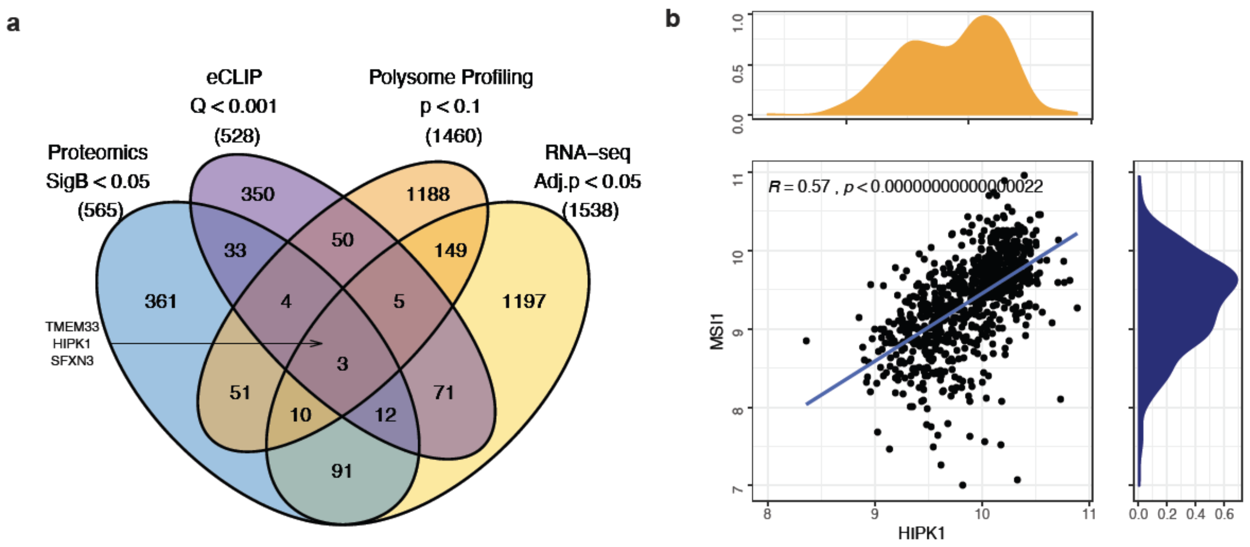

Supplementary Figure 9: Integrative analysis identifies a set of MSI1 downstream genes for potential therapeutic discovery with primary tumor abundance corroboration. **a.** Venn diagram showing convergence of TMEM33, SFNX3 and HIPK1 as significantly perturbed and MSI1 eCLIP-bound genes after sh*MSI1* inhibition, **b.** Illustrative figure of the diminishing number of significantly altered genes and their associated pathways with integrative analysis of multiple platforms in this study.

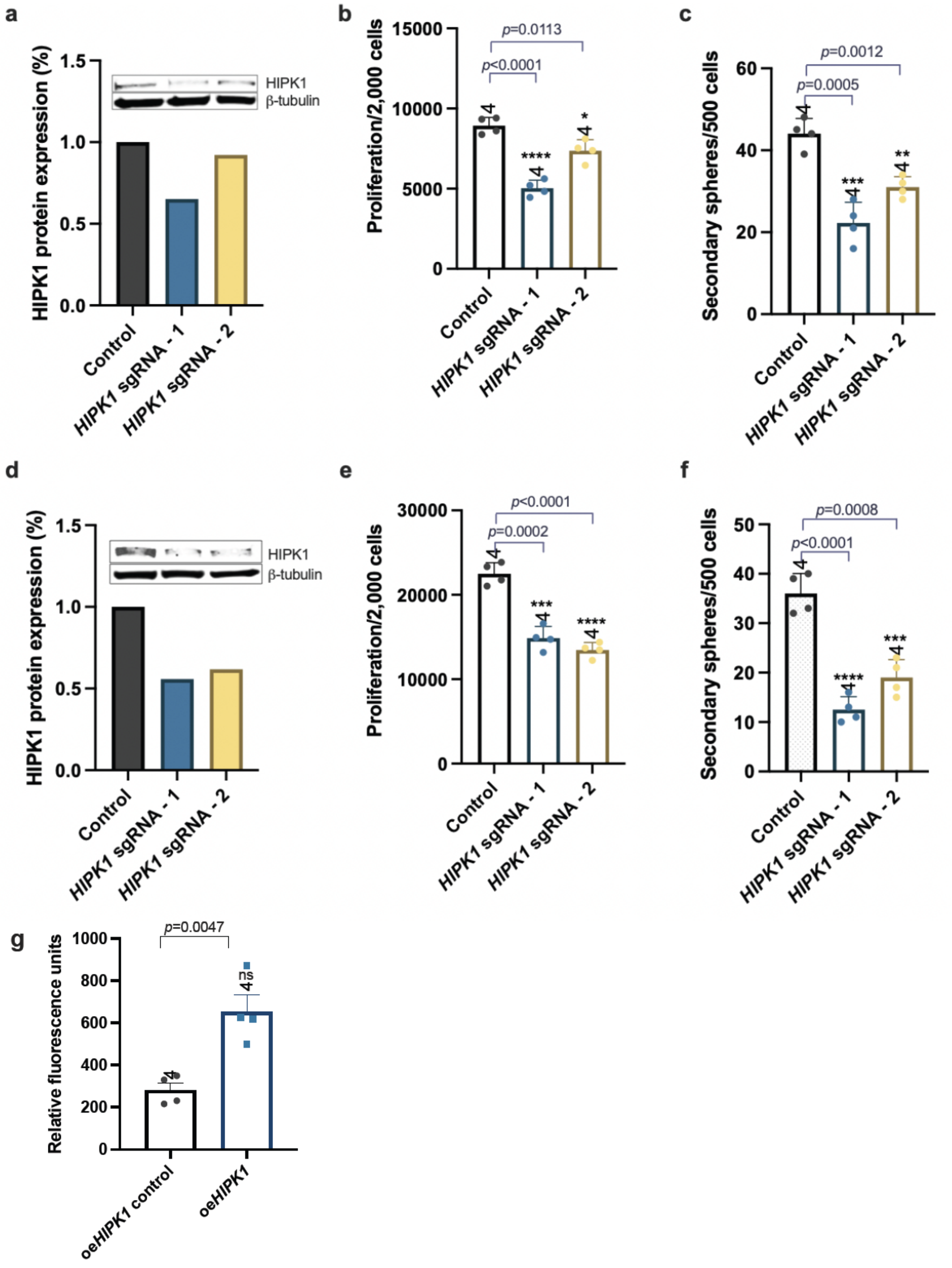

Supplementary Figure 10: HIPK1 CRISPR KO reduce stem cell properties in multiple G3 MB lines sparing NSCs with rescue of MSI1 KD with HIPK1 overexpression. a-c. SU_MB002 *HIPK1* polyclonal KO while modest reduction in expression, shows corresponding reduction in stem properties (for HIPK1 sgRNA-1, Proliferation: $p<0.0001$; Secondary sphere formation: $p=0.0005$). **d-f.** *HIPK1* polyclonal KO validation in another G3 MB cell line, HD-MB03 shows a similar functional affect (for HIPK1 sgRNA-1, Proliferation $p=<0.0002$; Secondary sphere formation $p<0.0001$**). h.** verification of shMSI1 KD and effect on proliferation in HD-MB03 ($p=0.0001$**). g.** *HIPK1* overexpression in HD-MB03 rescues the abrogation of stem cell properties after sh*MSI1* knockdown ($p=0.0047$).

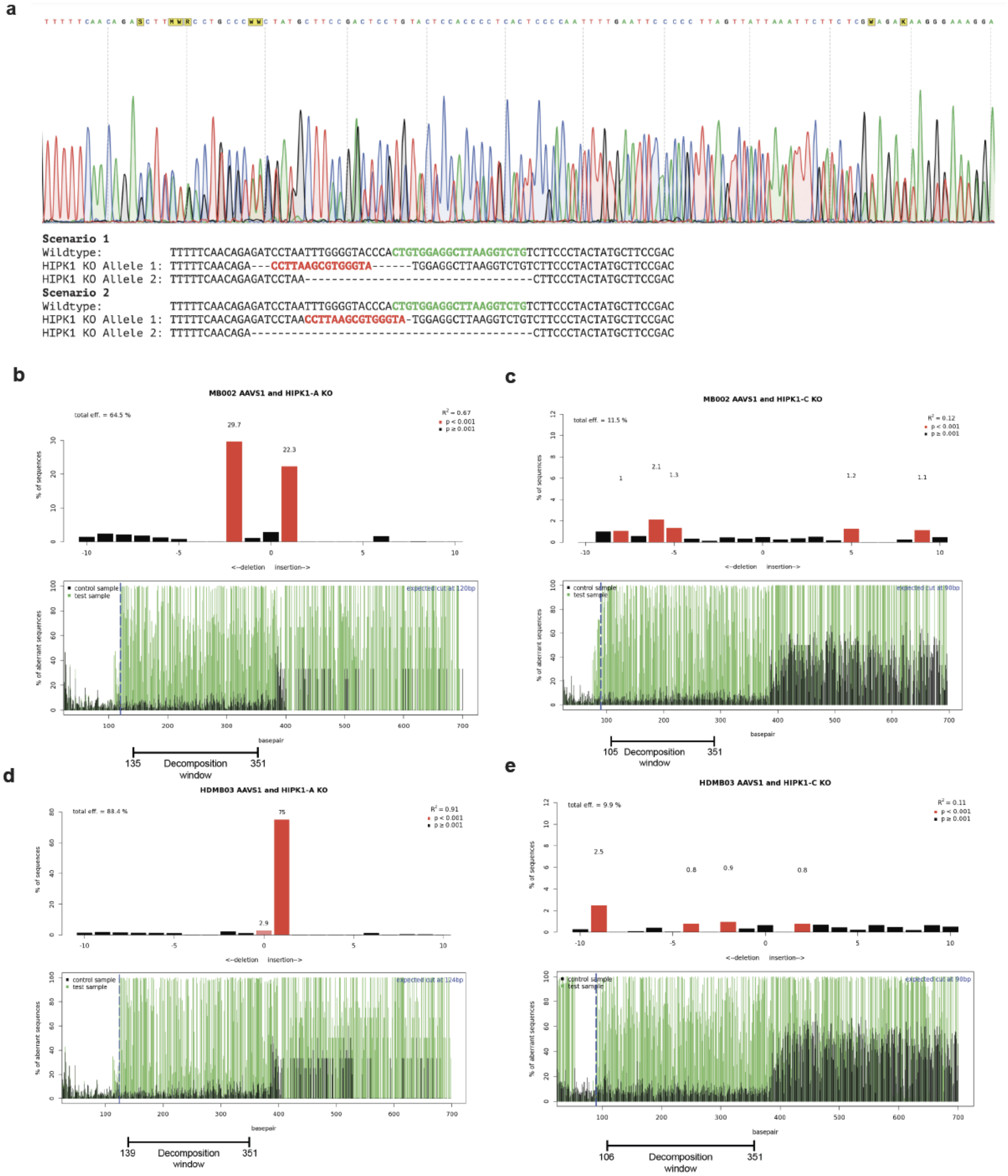

Supplementary Figure 11 HIPK1 CRISPR KO deletion: **a.** Sanger sequencing chromatogram of SU_MB002 and HD-MB03 cells with isogenic HIPK1 knockout with confirmation of AAVS (control) KO of wild-type genotype (top) with interpreted allele sequences (bottom). HIPK1-targeting sgRNA sequence shown in green, inserted sequences are shown in red, and deletions as dashes. **b-e.** TIDE (Tracking of Indels by Decomposition) analysis of all insertions and deletions (indels) in HD-MB03 (A-B) or MB002 (C-D) tumor cells with *HIPK1* sgRNA A (A, C) or C (B, D) isogenic knockout as compared to *AAVS1* control. The indel spectrum plots (top) represent indels to explain the composite sequence trace. A goodness of fit test (R squared) and corresponding statistical significance value for each indel is displayed. The percentage of aberrant nucleotides (bottom) are plotted along the sequence trace of the control (*AAVS1*) or experimental (*HIPK1*) samples.

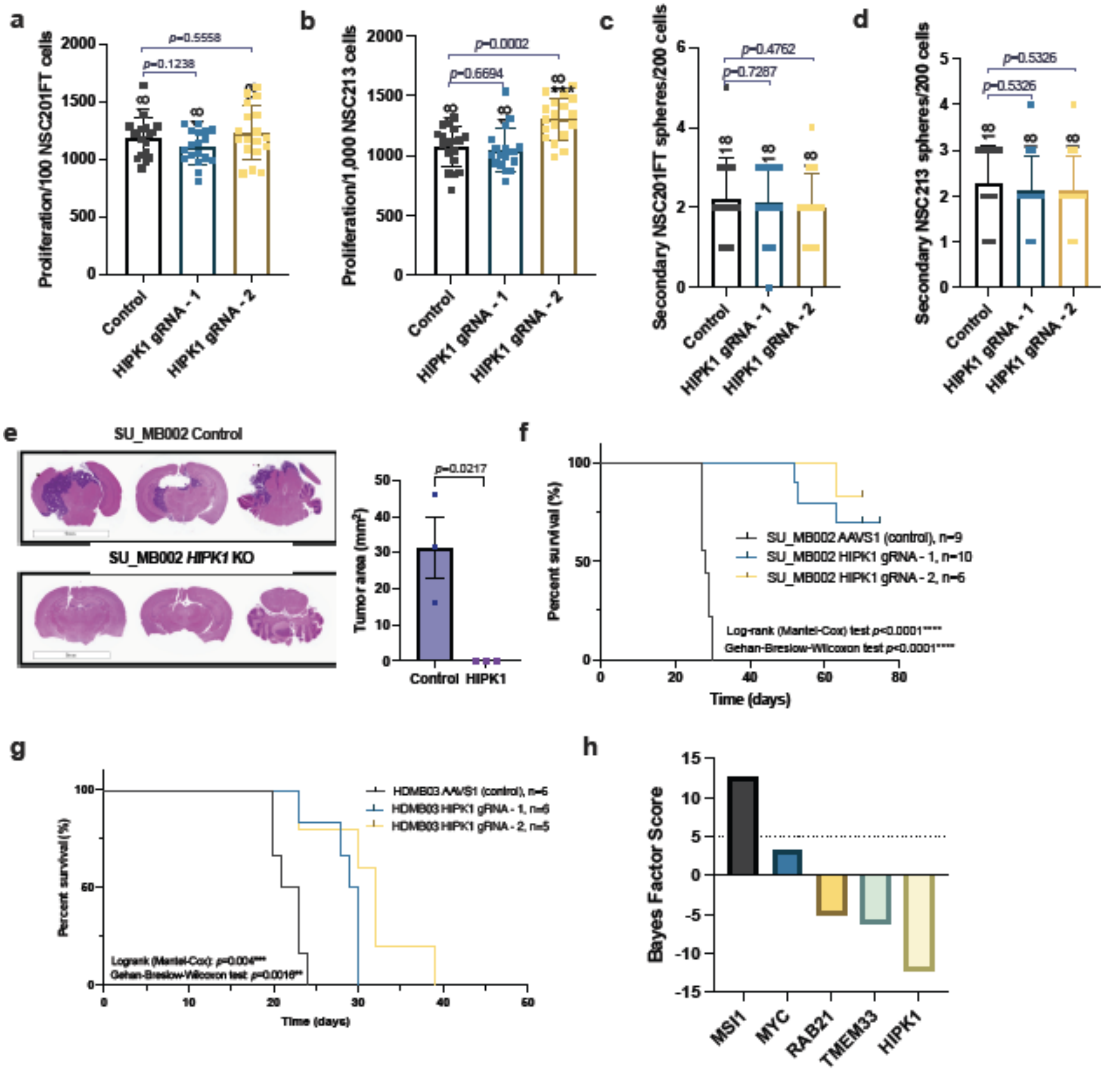

Supplementary Figure 12 HIPK1 CRISPR KO spares human NSC stem cell function validating the MSI1 multi-platform approach to targeted drug discovery. a-b. HIPK1 KO shows insignificant change in proliferation as compared to control in both NSC201FT (*p*=0.5558) and NSC213 (*p*=0.6694), **c-d.** HIPK1 KO shows insignificant change in secondary sphere formation as compared to control in both NSC201FT (*p*=0.7287) and NSC213 (*p*=0.5326), NSC213 *HIPK1* polyclonal KO shows limited difference in proliferation (*p*=0.4608), **e-g.** *in vivo* injections of clonal *HIPK1* KO shows significant reduction in tumor burden, (*p*=0.0217) and a striking survival benefit in 2 isogenic HIPK1 knockout cells that were clonally expanded in both SU_MB002 ((*p*<0.0001) and HDBM03 (*p*=0.004), **h.** Toledo *et al* [2]genome-wide CRISPR-Cas9 screen revealing essentiality showing essentiality of MSI1 compared to HIPK1 in NSCs (BF>5 corresponding to FDR<0.0

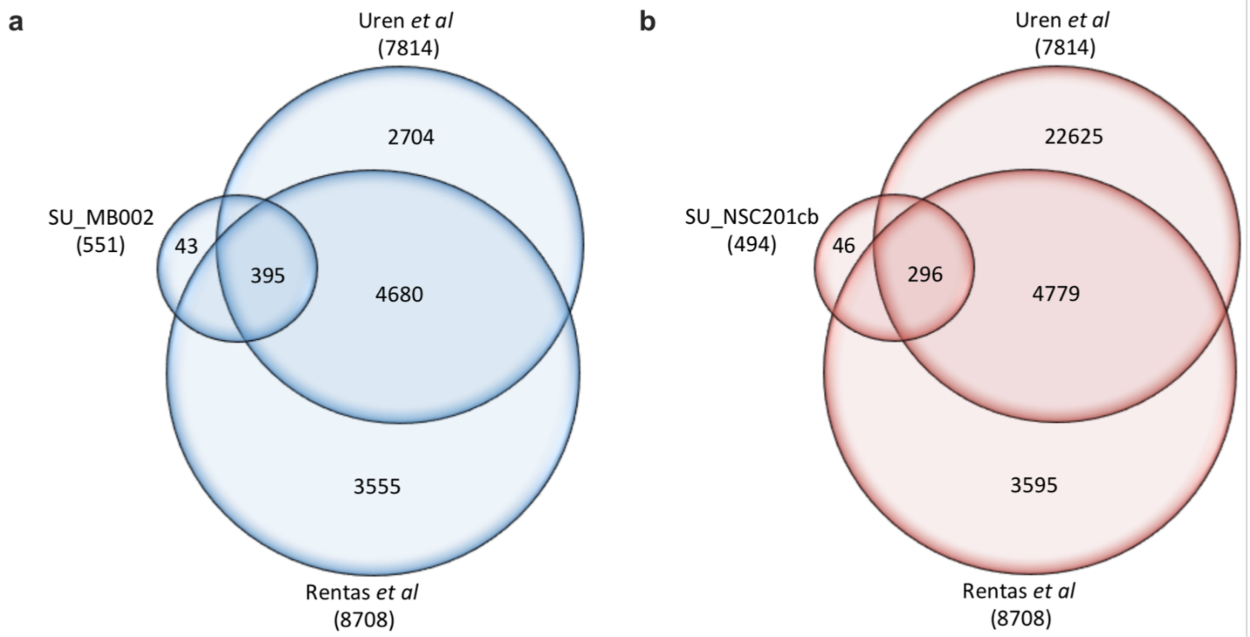

Supplementary Figure 13: Comparative analysis of SU_MB002 and NSC201cb MSI1-eCLIP showing considerable overlap of genes bound to MSI. a. Venn diagram of SI_MB002 MSI1 eCLIP targets compared to MSI1 iCLIP targets in glioblastoma from Uren *et al* [3]and MSI2 in HSPC from Rentas *et al*[4] (>99.9%), **b.** Venn diagram of NSC201cb MSI1 eCLIP targets compared to MSI1 iCLIP targets in glioblastoma from Uren *et al*[3] and MSI2 in HSPC from Rentas *et al*[4] (>99.9%).

a

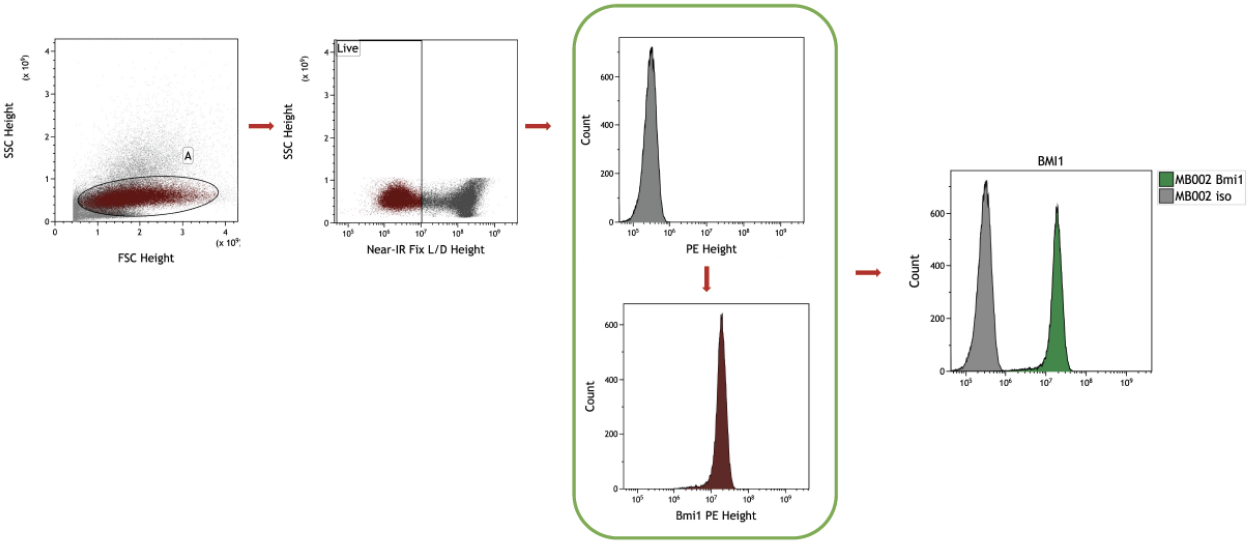

b

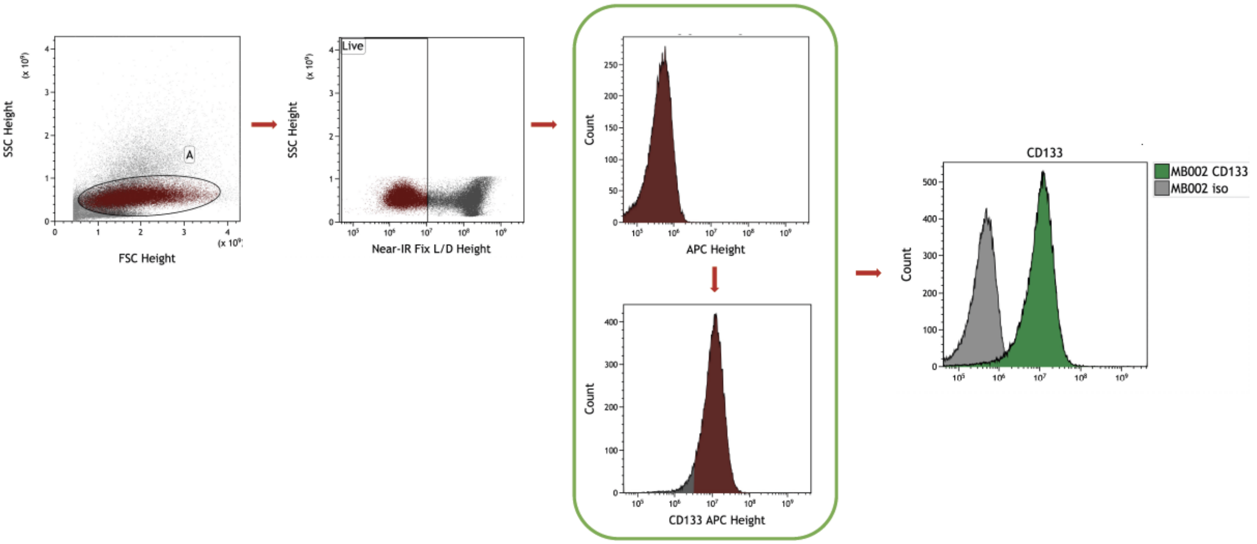

Supplementary Figure 14: Flow cytometry gating strategy **a. Gating strategy for CD133 and b. BMI1.**

References

1 Pei, Y. *et al.* An animal model of MYC-driven medulloblastoma. *Cancer Cell* **21**, 155-167, doi:10.1016/j.ccr.2011.12.021 (2012).

2 Toledo, C. M. *et al.* Genome-wide CRISPR-Cas9 Screens Reveal Loss of Redundancy between PKMYT1 and WEE1 in Glioblastoma Stem-like Cells. *Cell Rep* **13**, 2425-2439, doi:10.1016/j.celrep.2015.11.021 (2015).

3 Uren, P. J. *et al.* RNA-Binding Protein Musashi1 Is a Central Regulator of Adhesion Pathways in Glioblastoma. *Mol Cell Biol* **35**, 2965-2978, doi:10.1128/MCB.00410-15 (2015).

4 Rentas, S. *et al.* Musashi-2 attenuates AHR signalling to expand human haematopoietic stem cells. *Nature* **532**, 508-511, doi:10.1038/nature17665 (2016).

Supplementary tables

Supplementary Table 1: Top 50 reproducible peaks per IDR identified and quantified in SU_MB002

Chromosome	$Log_{20}p$	log_2FC	Strand	Gene ID	Gene name	Binding location
chr12	130.9069785	8.777033777	+	ENSG00000111678.6	C12orf57	5utr
chr12	85.29091126	7.902410526	-	ENSG00000060982.10	BCAT1	3utr
chr5	81.07294875	6.837477208	+	ENSG00000015479.13	MATR3	3utr
chr20	59.91804093	5.840674085	-	ENSG00000200494.1	Y_RNA	noncoding exon
chr16	52.57756841	7.25922924	-	ENSG00000170540.10	ARL6IP1	3utr
chr7	43.64426868	6.247806948	+	ENSG00000242265.1	PEG10	3utr
chr1	42.91109846	5.029559045	-	ENSG00000173726.6	TOMM20	3utr
chrM	42.58447578	5.321081504	-	ENSG00000210196.2	MT-TP	noncoding exon
chr12	40.45456608	6.985242013	+	ENSG00000179195.11	ZNF664	3utr
chr4	38.53043893	5.423984001	-	ENSG00000118816.5	CCNI	3utr
chr11	37.7841246	3.556087385	+	ENSG00000251562.3	MALAT1	noncoding exon
chr21	37.58937087	6.769725775	+	ENSG00000198743.5|| ENSG00000272962.1	SLC5A3	3utr
chr12	37.534259	6.783979979	+	ENSG00000149948.9	HMGA2	3utr
chr12	36.87039582	4.937627988	+	ENSG00000135486.13	HNRNPA1	3utr
chr15	35.15934931	6.779854277	-	ENSG00000140391.10	TSPAN3	3utr
chrX	34.96593011	5.784971341	+	ENSG00000102144.9	PGK1	3utr
chr16	34.59372627	5.786678363	+	ENSG00000091651.4	ORC6	3utr
chr15	33.25813174	4.433604362	-	ENSG00000247809.3	NR2F2-AS1	noncoding exon
chr12	33.05641151	4.857046447	+	ENSG00000008394.8	MGST1	3utr
chr3	32.55258876	5.055059351	+	ENSG00000156976.10	EIF4A2	3utr
chr17	32.18120884	6.646291422	-	ENSG00000136450.8	SRSF1	3utr
chr6	31.84807882	5.022863505	+	ENSG00000196230.8	TUBB	3utr
chrX	31.5259295	4.410543501	+	ENSG00000147140.11	NONO	3utr
chrX	29.91107758	5.51403978	+	ENSG00000131171.8	SH3BGRL	3utr
chr10	29.71255077	5.561559347	+	ENSG00000099194.5	SCD	3utr
chr11	29.59140392	4.938285787	-	ENSG00000110321.11	EIF4G2	3utr
chrM	28.99250656	6.49170087	+	ENSG00000210164.1	MT-TG	noncoding exon
chr2	27.53936702	5.455572826	+	ENSG00000176887.5	SOX11	3utr
chr11	26.79778259	4.871195681	-	ENSG00000152558.10	TMEM123	3utr
chr14	25.96881056	4.777721156	-	ENSG00000174373.11	RALGAPA1	3utr
chr15	25.84084233	6.339717622	-	ENSG00000140319.6	SRP14	3utr
chr16	25.65569263	6.375022115	+	ENSG00000230989.2	HSBP1	3utr
chrX	25.61407125	5.406349608	+	ENSG00000102144.9	PGK1	3utr
chr22	24.90027276	6.23451097	+	ENSG00000189060.4	H1F0	3utr
chr12	24.83009878	5.384932835	-	ENSG00000187109.9	NAP1L1	3utr
chr7	24.31613848	6.244869532	-	ENSG00000105810.5	CDK6	3utr
chr11	24.0579986	4.941801413	+	ENSG00000134333.9	LDHA	3utr
chr14	23.33098965	4.727164562	-	ENSG00000080824.14	HSP90AA1	3utr
chr2	23.29552509	6.115726965	+	ENSG00000170144.14	HNRNPA3	3utr
chr8	23.23874893	3.352849272	-	ENSG00000070756.9	PABPC1	CDS
chr14	22.97695342	4.21446932	+	ENSG00000100941.4	PNN	CDS
chr6	22.94225784	3.025370111	+	ENSG00000112306.7	RPS12	CDS
chr17	22.91814631	6.284106912	+	ENSG00000129657.10	SEC14L1	3utr
chr2	22.11627158	4.257377881	-	ENSG00000115758.8	ODC1	3utr
chr16	21.94445387	6.010600185	-	ENSG00000167005.9	NUDT21	3utr
chr22	21.89832131	4.292426182	-	ENSG00000184117.7	NIPSNAP1	3utr
chr10	20.96114468	5.994796425	+	ENSG00000148798.5	INA	3utr
chr5	20.57943831	4.990571912	+	ENSG00000131507.9	NDFIP1	3utr
chr18	20.56341885	5.887381237	+	ENSG00000101557.10	USP14	3utr

Supplementary Table 2: Top 50 of reproducible peaks per IDR identified and quantified in NSC201cb

Chromosome	$log_{10}p$	log2FC	Strand	Gene ID	Gene name	Binding location
chr13	217.7647656	8.017232101	+	ENSG00000136156.8	ITM2B	3utr
chr3	114.1054669	7.135879065	+	ENSG00000044524.6	EPHA3	3utr
chr21	112.7230695	7.091495696	-	ENSG00000142192.16	APP	3utr
chr16	92.69099682	7.539088307	-	ENSG00000170540.10	ARL6IP1	3utr
chr17	90.75969817	6.398451659	+	ENSG00000129657.10	SEC14L1	3utr, CDS
chrX	85.6133828	6.297131384	-	ENSG00000046653.10	GPM6B	3utr, CDS
chr3	85.25096298	6.689735973	+	ENSG00000044524.6	EPHA3	3utr
chr5	84.74867819	4.711242582	-	ENSG00000113140.6	SPARC	3utr
chr10	84.74438984	5.014445832	+	ENSG00000026025.9	VIM	3utr
chr5	84.17973284	4.621682212	+	ENSG00000127022.10	CANX	3utr
chr1	83.22261668	4.31953998	-	ENSG00000132688.10	NES	3utr
chr10	80.08848551	3.836734099	-	ENSG00000197746.9	PSAP	3utr
chr10	78.04203853	3.921922292	+	ENSG00000099194.5	SCD	3utr
chr4	76.29918028	6.849455618	-	ENSG00000150625.12	GPM6A	3utr
chr2	74.17232709	3.208316853	-	ENSG00000115461.4	IGFBP5	3utr
chr7	74.07030771	8.019971227	+	ENSG00000106278.7	PTPRZ1	3utr
chr17	72.02050626	4.184159196	-	ENSG00000035862.8	TIMP2	3utr
chr16	66.89720728	6.438424985	+	ENSG00000087245.8	MMP2	3utr
chr5	64.89389141	7.499908527	+	ENSG00000131507.9	NDFIP1	3utr
chr2	64.76386598	5.371633971	-	ENSG00000115310.13	RTN4	3utr
chr4	62.57174239	7.757839672	-	ENSG00000145284.7	SCD5	3utr
chr2	59.90751481	6.499205975	+	ENSG00000138448.7	ITGAV	3utr
chr3	57.7510219	6.440111785	-	ENSG00000249992.1	TMEM158	3utr
chr7	56.37072295	4.367158893	-	ENSG00000146674.10	IGFBP3	3utr
chr4	56.36812144	7.620212461	-	ENSG00000150625.12	GPM6A	3utr
chr5	55.42376234	3.673434928	-	ENSG00000019582.10	CD74	3utr
chr5	55.04095317	6.93175982	+	ENSG00000131711.10	MAP1B	3utr
chr4	53.51981046	5.968547023	+	ENSG00000118785.9	SPP1	3utr
chrX	53.24762415	6.144752641	+	ENSG00000071553.12	ATP6AP1	3utr
chr8	52.25320783	7.709703391	+	ENSG00000168615.7	ADAM9	3utr
chr6	52.17422996	3.019355171	+	ENSG00000204580.7	DDR1	3utr
chr15	51.0747137	4.097832924	-	ENSG00000067225.13	PKM	3utr
chr5	49.88087911	6.489699082	+	ENSG00000131711.10	MAP1B	3utr
chr8	49.5919976	4.691394721	-	ENSG00000164733.16	CTSB	3utr
chr15	48.98493379	4.106230029	+	ENSG00000166710.13	B2M	3utr
chr16	48.85010379	4.952091381	+	ENSG00000087245.8	MMP2	3utr
chr12	48.18395494	5.745688464	+	ENSG00000174437.12	ATP2A2	3utr CDS
chr12	47.29393116	4.155004143	+	ENSG00000111640.10	GAPDH	3utr
chr15	46.05393111	4.025920892	-	ENSG00000067225.13	PKM	3utr
chr1	45.7614308	7.389914631	+	ENSG00000117600.8	LPPR4	3utr
chr2	43.85614242	5.036419588	+	ENSG00000213639.5	PPP1CB	3utr
chr11	42.72397501	3.390281252	+	ENSG00000134824.9	FADS2	3utr
chr6	40.54225356	6.030715387	-	ENSG00000112276.9	BVES	3utr
chr10	40.29638693	5.680760829	+	ENSG00000107984.5	DKK1	3utr
chr1	39.7511933	4.808361811	-	ENSG00000171603.12	CLSTN1	3utr
chr5	39.74508304	7.120078512	-	ENSG00000171617.9	ENC1	3utr
chr14	39.21487853	6.1380383	-	ENSG00000080824.14	HSP90AA1	3utr
chr17	38.6256614	5.395194207	-	ENSG00000131095.7	GFAP	3utr
chr18	38.19222827	3.891465185	-	ENSG00000170558.4	CDH2	3utr
chr3	37.00605891	5.801302771	+	ENSG00000168036.12	CTNNB1	3utr

Supplementary Table 3: Shared genes between SU_MB002 and NSC201cb (N=110)

GENE ID	
ACTR2	NHLRC2
ANP32A	NONO
AP2B1	NORAD
APP	NUCKS1
ARL6IP1	NUFIP2
ARPP19	NXN
ATP2A2	PARP1
ATP6AP2	PFN2
ATP6V1G1	PGAM1
ATPAF1	PGK1
AZIN1	PPP1CB
CADM1	PREPL
CALD1	PRKAR1A
CALM1	PTPRG
CALU	PTTG1IP
CANX	RAB11A
CCNI	RAC1
CDC42	RGMB
CDK6	RNF187
CKAP5	RPN2
CKB	RTN4
CLIC4	SCARB2
CLSTN1	SCD
CTNNB1	SEC14L1
DCBLD2	SEPT11
DDAH2	SERINC1
DRAP1	SESN3
DYNLL2	SFXN3
EIF4A2	SLC16A1
EIF4G2	SLC38A2
ELOVL6	SLC39A14
EPT1	SLC39A6
FAM168B	SLC5A3
FSCN1	SON
GLUL	SPCS1
GNG12	SPCS3
GSE1	SRP14
GSPT1	STOM
HIPK2	TCF12
HMGCS1	TMBIM6
HNRNPU	TMED7
HSP90AA1	TMEM123
IGF1R	TMEM248
LAPTM4B	TNPO1
LDHA	TOMM20
LIPA	TRIM9
MAP1B	TROVE2
MARCH6	TSPAN3
MARCKS	TTC3
MARCKSL1	TUBB
MLEC	WNK1
NARS	YWHAG
NCKAP1	ZDHHC9
NDFIP1	ZNF106
NGRN	ZNF664

Supplementary Table 4: MSI1 eCLIP binding region counts for NSC201cb and SU_MB002

Binding Region	NSC201cb	SU_MB002
Coding Sequence	2979	5021
Distal intron	3994	7842
Proximal intron	1407	1178
5' UTR	173	160
3' UTR	68795	83213
Uncategorized	678	1506

Supplementary Table 5: Associated MSI1 eCLIP bound gene pathways

Term ID	Pathway	*p*-value	NSC201cb	SU_MB002
GO:0006397	mRNA processing	3.96E-13	0	1
GO:0008380	RNA splicing	4.25E-13	0	1
GO:0043062	extracellular structure organization	4.57E-13	1	0
GO:0000377	RNA splicing, via transesterification reactions with bulged adenosine as nucleophile	1.65E-12	0	1
GO:0000398	mRNA splicing, via spliceosome	1.65E-12	0	1
GO:0030198	extracellular matrix organization	2.29E-12	1	0
GO:0000375	RNA splicing, via transesterification reactions	2.30E-12	0	1
REAC:R-HSA-72163	mRNA Splicing - Major Pathway	4.75E-10	0	1
REAC:R-HSA-72172	mRNA Splicing	1.43E-09	0	1
GO:0050839	cell adhesion molecule binding	4.04E-09	1	0
REAC:R-HSA-72203	Processing of Capped Intron-Containing Pre-mRNA	5.68E-09	0	1
GO:0098589	membrane region	1.53E-08	1	0
GO:0044437	vacuolar part	3.20E-08	1	0
GO:0098857	membrane microdomain	4.85E-08	1	0
GO:1903311	regulation of mRNA metabolic process	5.01E-08	0	1
GO:0060284	regulation of cell development	5.06E-08	1	0
REAC:R-HSA-1474244	Extracellular matrix organization	5.54E-08	1	0
GO:0034655	nucleobase-containing compound catabolic process	6.23E-08	0	1
GO:0031252	cell leading edge	6.62E-08	1	0
GO:0006401	RNA catabolic process	8.55E-08	0	1
GO:0006402	mRNA catabolic process	1.23E-07	0	1
GO:0006403	RNA localization	1.95E-07	0	1
GO:0045121	membrane raft	2.71E-07	1	0
GO:0050767	regulation of neurogenesis	3.55E-07	1	0
GO:0030175	filopodium	4.40E-07	1	0
GO:0006413	translational initiation	5.21E-07	0	1
GO:0006417	regulation of translation	8.23E-07	0	1
GO:0051960	regulation of nervous system development	1.16E-06	1	0
GO:0031589	cell-substrate adhesion	1.61E-06	1	0
GO:0043312	neutrophil degranulation	2.23E-06	1	0
GO:0030667	secretory granule membrane	2.46E-06	1	0
GO:0002283	neutrophil activation involved in immune response	2.64E-06	1	0
GO:0036464	cytoplasmic ribonucleoprotein granule	2.75E-06	0	1
GO:0042119	neutrophil activation	4.13E-06	1	0
GO:0002446	neutrophil mediated immunity	4.37E-06	1	0
GO:0000904	cell morphogenesis involved in differentiation	5.22E-06	1	0
GO:0006446	regulation of translational initiation	5.62E-06	0	1
GO:0043202	lysosomal lumen	5.71E-06	1	0
GO:0036230	granulocyte activation	5.74E-06	1	0
GO:0034248	regulation of cellular amide metabolic process	7.60E-06	0	1
GO:0035770	ribonucleoprotein granule	8.79E-06	0	1
GO:0005775	vacuolar lumen	9.33E-06	1	0
GO:0071826	ribonucleoprotein complex subunit organization	1.21E-05	0	1
GO:0022618	ribonucleoprotein complex assembly	1.73E-05	0	1
GO:0031344	regulation of cell projection organization	1.90E-05	1	0
REAC:R-HSA-9006934	Signaling by Receptor Tyrosine Kinases	2.04E-05	1	0
GO:0050657	nucleic acid transport	2.06E-05	0	1
GO:0050658	RNA transport	2.06E-05	0	1
GO:0070937	CRD-mediated mRNA stability complex	2.10E-05	0	1
GO:0003727	single-stranded RNA binding	2.10E-05	0	1
GO:0042063	gliogenesis	2.12E-05	1	0
GO:0043487	regulation of RNA stability	2.34E-05	0	1

GO:0120039	plasma membrane bounded cell projection morphogenesis	2.40E-05	1	0
GO:0048858	cell projection morphogenesis	2.86E-05	1	0
GO:0051236	establishment of RNA localization	2.87E-05	0	1
GO:0098852	lytic vacuole membrane	2.88E-05	1	0
GO:0005765	lysosomal membrane	2.88E-05	1	0
GO:0015629	actin cytoskeleton	3.09E-05	1	0
GO:0120035	regulation of plasma membrane bounded cell projection organization	3.13E-05	1	0
GO:0031124	mRNA 3'-end processing	3.34E-05	0	1
GO:0098858	actin-based cell projection	3.46E-05	1	0
GO:0005788	endoplasmic reticulum lumen	3.50E-05	1	0
GO:0010494	cytoplasmic stress granule	3.76E-05	0	1
GO:0015931	nucleobase-containing compound transport	4.71E-05	0	1
REAC:R-HSA-8957275	Post-translational protein phosphorylation	4.72E-05	1	0
GO:0032990	cell part morphogenesis	5.05E-05	1	0
GO:0043488	regulation of mRNA stability	5.23E-05	0	1
GO:0031123	RNA 3'-end processing	5.48E-05	0	1
GO:0006913	nucleocytoplasmic transport	5.49E-05	0	1
REAC:R-HSA-6798695	Neutrophil degranulation	6.55E-05	1	0
GO:0043235	receptor complex	6.64E-05	1	0
GO:0030027	lamellipodium	6.77E-05	1	0
GO:0051169	nuclear transport	6.82E-05	0	1
REAC:R-HSA-381426	Regulation of Insulin-like Growth Factor (IGF) transport and uptake by Insulin-like Growth Factor Binding Proteins	6.82E-05	1	0
GO:0030424	axon	8.26E-05	1	0
GO:0006405	RNA export from nucleus	9.93E-05	0	1
GO:0098797	plasma membrane protein complex	0.000103	1	0
GO:0005518	collagen binding	0.000108	1	0
GO:0051592	response to calcium ion	0.000115	1	0
GO:0071277	cellular response to calcium ion	0.000126	1	0
REAC:R-HSA-72766	Translation	0.000138	0	1
GO:0048812	neuron projection morphogenesis	0.00015	1	0
GO:0005635	nuclear envelope	0.000153	0	1
GO:0030133	transport vesicle	0.000153	1	0
GO:0007160	cell-matrix adhesion	0.000163	1	0
GO:0000785	chromatin	0.000172	0	1
GO:0005774	vacuolar membrane	0.000234	1	0
GO:0051052	regulation of DNA metabolic process	0.00026	0	1
REAC:R-HSA-216083	Integrin cell surface interactions	0.000274	1	0
GO:0046916	cellular transition metal ion homeostasis	0.000279	0	1
GO:0033627	cell adhesion mediated by integrin	0.000288	1	0
REAC:R-HSA-2682334	EPH-Ephrin signaling	0.000289	1	0
GO:0061564	axon development	0.000291	1	0
GO:0072594	establishment of protein localization to organelle	0.000315	0	1
GO:0071564	npBAF complex	0.000316	0	1
REAC:R-HSA-4420097	VEGFA-VEGFR2 Pathway	0.00032	1	0
GO:0030155	regulation of cell adhesion	0.000366	1	0
GO:1903293	phosphatase complex	0.000369	0	1
GO:0008287	protein serine/threonine phosphatase complex	0.000369	0	1
GO:0050840	extracellular matrix binding	0.000435	1	0
GO:0034330	cell junction organization	0.000439	1	0
REAC:R-HSA-373755	Semaphorin interactions	0.000461	1	0
GO:0071013	catalytic step 2 spliceosome	0.000462	0	1
GO:0045664	regulation of neuron differentiation	0.000526	1	0

GO:0032870	cellular response to hormone stimulus	0.000581	1	0
REAC:R-HSA-195258	RHO GTPase Effectors	0.000595	1	1
GO:0031346	positive regulation of cell projection organization	0.00061	1	0
GO:0009611	response to wounding	0.000629	1	0
GO:0061013	regulation of mRNA catabolic process	0.000649	0	1
REAC:R-HSA-194138	Signaling by VEGF	0.000678	1	0
GO:0045296	cadherin binding	0.00073	1	1
GO:0031258	lamellipodium membrane	0.00081	1	0
GO:0042382	paraspeckles	0.000853	0	1
GO:0060627	regulation of vesicle-mediated transport	0.000863	1	0
GO:0097435	supramolecular fiber organization	0.000882	1	0
GO:0055076	transition metal ion homeostasis	0.000933	0	1
GO:0048667	cell morphogenesis involved in neuron differentiation	0.000936	1	0
GO:0032241	positive regulation of nucleobase-containing compound transport	0.00103	0	1
GO:0046833	positive regulation of RNA export from nucleus	0.00103	0	1
GO:0070934	CRD-mediated mRNA stabilization	0.00103	0	1
REAC:R-HSA-114452	Activation of BH3-only proteins	0.00103	0	1
REAC:R-HSA-5628897	TP53 Regulates Metabolic Genes	0.00104	0	1
GO:0051168	nuclear export	0.00109	0	1
GO:0003682	chromatin binding	0.00112	0	1
GO:0070161	anchoring junction	0.00123	1	1
GO:1903312	negative regulation of mRNA metabolic process	0.00126	0	1
GO:0043489	RNA stabilization	0.00133	0	1
GO:0007409	axonogenesis	0.00133	1	0
GO:0120034	positive regulation of plasma membrane bounded cell projection assembly	0.00136	1	0
REAC:R-HSA-156827	L13a-mediated translational silencing of Ceruloplasmin expression	0.00137	0	1
GO:0032204	regulation of telomere maintenance	0.00147	0	1
REAC:R-HSA-5627123	RHO GTPases activate PAKs	0.00147	1	0
GO:0044770	cell cycle phase transition	0.00148	0	1
GO:0098687	chromosomal region	0.00148	0	1
GO:0060560	developmental growth involved in morphogenesis	0.00169	1	0
GO:0010720	positive regulation of cell development	0.00171	1	0
GO:0000139	Golgi membrane	0.00172	1	0
GO:0098553	lumenal side of endoplasmic reticulum membrane	0.00172	1	0
GO:0071556	integral component of lumenal side of endoplasmic reticulum membrane	0.00172	1	0
GO:0005911	cell-cell junction	0.00175	1	0
GO:0051491	positive regulation of filopodium assembly	0.00179	1	0
GO:0008143	poly(A) binding	0.00185	0	1
GO:0070820	tertiary granule	0.00185	1	0
GO:0030658	transport vesicle membrane	0.00189	1	0
GO:0005793	endoplasmic reticulum-Golgi intermediate compartment	0.00192	1	0
REAC:R-HSA-6803529	FGFR2 alternative splicing	0.00193	0	1
GO:0033116	endoplasmic reticulum-Golgi intermediate compartment membrane	0.00197	1	0

Supplementary Table 6: Pathway analysis of SU_MB002 MSI1-bound genes associated with chromatin remodeling, transcription and translation, JAK-STAT and TGF-β pathways

Chromatin, Transcription, Translation						JAK-STAT	TGF- β
ACTR2	DCP2	HLTF	MCM4	PTBP3	SQLE	CANX	CCNT1
AGO1	DCTN4	HMGA2	MDN1	PTGES3	SRP14	CDC42	DRAP1
AKIRIN1	DCTN5	HMGB2	MORF4L2	PTTG1IP	SRSF1	HNRNPA2B1	FKBP1A
ANP32A	DCTPP1	HMGCR	MPZL1	RAB11A	SRSF2	HNRNPDL	MYC
ANP32E	DDX1	HMGCS1	MRPL42	RAB1A	SRSF6	HNRNPF	PPP1CB
APP	DDX3X	HMGN1	MRPL48	RAB21	SSR3	HSPA9	PPP1CC
ARHGAP42	DEK	HNRNPA0	MRPS28	RAC1	SSU72	PAK2	TFDP2
ARHGEF12	DHX9	HNRNPA1	MRPS30	RACGAP1	STAMBP	SOD1	TGFBR1
ARID1A	DKC1	HNRNPA2B1	MTPN	RAD23B	STMN1		
ARL6IP1	DNAJC8	HNRNPA3	MXI1	RAN	STRAP		
ARPP19	DPYSL2	HNRNPD	MYC	RBBP4	SUMO2		
ATP6V1G1	DR1	HNRNPDL	NARS	RBFOX2	SYCP2L		
AUTS2	DRAP1	HNRNPF	NCBP2	RBM14	SYNCRIP		
AZIN1	DYNLL2	HNRNPH1	NCKAP1	RBMX	SYNE2		
BACH1	EID1	HNRNPH2	NDFIP1	RERE	TBL1XR1		
BASP1	EIF1AX	HNRNPH3	NELL2	RLIM	TCF12		
BAZ2A	EIF2A	HNRNPR	NEUROD1	RPL15	TFAM		
BCAT1	EIF2S1	HNRNPU	NGRN	RPL22L1	TFDP2		
BRD3	EIF2S3	HPRT1	NOLC1	RPL23A	TFRC		
BTBD1	EIF3E	HSBP1	NONO	RPL27A	TGFBR1		
BUB3	EIF4A2	HSP90AA1	NPM1	RPL37	TIMM13		
C12ORF57	EIF4B	HSPA4	NUCKS1	RPN2	TMBIM6		
CADM1	EIF4EBP2	HSPA9	NUDT21	RPS12	TMCO1		
CALD1	EIF4G2	IGF2BP1	NUFIP2	RPS23	TMEM33		
CALM1	ELAVL1	IGF2BP3	NUP155	RRM2	TMEM97		
CALM2	ELOVL5	INA	NUSAP1	RSL24D1	TMPO		
CAND1	ELOVL6	INCENP	ORC6	RTN4	TNPO1		
CANX	EMC4	INSIG1	OTX2	SCARB2	TOMM20		
CAPRIN1	ENAH	IPO7	PABPC1	SCD	TP53BP2		
CBFB	EPB41	JAM3	PAICS	SEC11A	TPM4		
CBX3	ERCC1	KHDRBS1	PAK2	SERP1	TRIM9		
CBX5	EXOC5	KIF5B	PAPOLA	SESN3	TUBA1B		
CCNB1IP1	FAM172A	KPNA4	PARP1	SF3B1	TUBB		
CCNG1	FHL1	KRAS	PAWR	SFPQ	TXNRD1		
CCNT1	FKBP1A	LAMTOR3	PCBP1	SKA2	U2AF1		
CCT4	FSCN1	LAMTOR5	PCBP2	SLBP	UBA52		
CCT5	FUBP1	LAPTM4B	PDLIM5	SLC25A36	UBE2J1		
CDC42	FYTTD1	LARP1	PFN2	SLC39A10	UNC119		
CDK6	G3BP1	LARP4	PGAM1	SLC39A14	UPF3B		
CDKN2A	GABARAPL2	LBR	PGK1	SLC39A6	URI1		
CENPF	GDI2	LDHA	PHF10	SMAD5	USP14		
CHERP	GLS	LIN28B	PNN	SMARCA4	USP33		
CKAP5	GNG12	LIN7C	POLR2D	SMARCC1	WAC		
CKB	GSPT1	LNPEP	PPA1	SMARCD1	WDR77		
CLIC4	GTF2F2	LONP2	PPP1CB	SMARCE1	WDR82		
CNBP	H1F0	LRPPRC	PPP1CC	SMC1A	XPOT		
CNOT6	H2AFV	MAD2L1	PPP1R15B	SMG1	XRCC5		
COX7C	H2AFY	MAGEF1	PPP2CA	SNX1	YWHAE		
CPSF6	H3F3B	MAP1B	PPP3R1	SOD1	YWHAG		
CSDE1	HDGF	MAP1LC3B	PPP4R2	SON	YWHAQ		
CTNNB1	HELLS	MAPRE1	PRKAR1A	SOX11	YWHAZ		
CUL3	HIPK1	MARCH6	PRKDC	SPCS1	ZBTB18		
CYCS	HIPK2	MARCKS	PRNP	SPCS3	ZNF638		
DAAM1	HK2	MCL1	PTBP2				

Supplementary Table 7: Pathway analysis of NSC201cb MSI1-bound genes associated with chromatin remodeling, transcription and translation pathways

**Chromatin, Transcription, Translation**
ADAM9
ANXA2
AZIN1
BCAP31
CSNK1D
GFAP
GNA12
HIPK2
HSP90AA1
HSP90AB1
LAPTM4B
MAP1A
MSN
OAZ2
PTTG1IP
RAB7A
RDX
SGTA
TIMP2
TMTC3
TRIB2

Supplementary Table 8: Top 50 transcript differentially expressed (FDR<0.05) in control vs sh*MSI1*-inhibited samples identified and quantified

Gene ID	logFC	*p*-value	adj. *p*-value
SAMD11	6.011459384	3.97E-15	2.26E-11
ID1	5.963583538	4.16E-15	2.26E-11
SMAD9	3.935721549	3.71E-13	1.34E-09
GNGT1	-3.292823676	2.19E-12	5.95E-09
FEZF1-AS1	-2.567671031	3.84E-12	8.33E-09
TNC	-2.310780904	1.24E-11	2.24E-08
DIAPH2-AS1	-2.26936437	1.96E-11	3.04E-08
VXN	-2.983732204	2.64E-11	3.21E-08
CCDC141	2.805275472	2.66E-11	3.21E-08
RXRG	2.692853253	4.27E-11	4.63E-08
KIRREL1	2.323398387	6.74E-11	6.64E-08
NID1	-3.25026725	7.77E-11	7.02E-08
GABRR3	-3.127691513	1.45E-10	1.21E-07
GPRC5B	3.721258921	4.78E-10	3.71E-07
ID3	4.378383972	5.76E-10	4.16E-07
C3orf70	-3.045293789	1.03E-09	6.97E-07
SMAD6	3.938520964	1.33E-09	8.46E-07
ST8SIA3	-2.876526013	1.52E-09	9.18E-07
EEF1A2	3.227050274	1.75E-09	1.00E-06
NEUROG1	2.342033954	1.94E-09	1.06E-06
RAB31	3.357439607	3.04E-09	1.57E-06
LIN28B	1.753633752	3.61E-09	1.68E-06
PRKCB	-3.989528579	3.67E-09	1.68E-06
TSPAN18	2.305888732	3.76E-09	1.68E-06
UCHL1	-3.37932154	3.87E-09	1.68E-06
CELF2	-3.212076214	4.84E-09	2.02E-06
ARSI	3.043440189	5.04E-09	2.03E-06
SOAT1	2.035855402	5.39E-09	2.09E-06
CRABP2	2.110811056	6.09E-09	2.28E-06
KIAA2012	2.702318067	6.69E-09	2.42E-06
SYNPR	2.937504115	8.58E-09	3.00E-06
DOCK8	2.180837079	9.22E-09	3.13E-06
TRIM55	-1.934371715	9.79E-09	3.22E-06
MCTP1	-2.790540676	1.07E-08	3.41E-06
SPAG1	-2.016176018	1.17E-08	3.64E-06
LIMCH1	2.327055316	1.76E-08	5.29E-06
REST	1.929339389	3.65E-08	1.07E-05
ETV3L	3.403079006	6.23E-08	1.78E-05
NRL	2.973061278	9.05E-08	2.52E-05
RALGAPA1	-1.936989934	1.11E-07	3.00E-05
PTPN13	1.917199568	1.14E-07	3.00E-05
B4GALT1	2.399457238	1.29E-07	3.32E-05
CNTN1	-1.942528026	1.40E-07	3.50E-05
NOX5	-1.78678262	1.42E-07	3.50E-05
BASP1	-1.574192654	1.84E-07	4.43E-05
SMYD1	-2.462259577	2.35E-07	5.55E-05
MIOS	1.508871056	3.09E-07	7.12E-05
PCDH10	-2.571616122	3.93E-07	8.71E-05
CHST2	2.418298282	3.93E-07	8.71E-05
IFI44	-2.264241404	4.26E-07	9.08E-05

Supplementary Table 9: SU_MB002 and NSC201cb MSI1 eCLIP bound gene annotated as an RNA binding protein (RBP)

Gene ID	Baltz *et al*	Castello *et al*	SU_MB002	NSC201cb
ANP32A	1	0	1	1
CANX	1	1	1	1
EIF4G2	1	1	1	1
EIF4A2	1	1	1	1
FSCN1	0	1	1	1
GSPT1	1	1	1	1
HNRNPU	1	1	1	1
HSP90AA1	1	1	1	1
NGRN	1	1	1	1
NONO	1	1	1	1
NUCKS1	1	0	1	1
NUFIP2	1	1	1	1
PARP1	1	1	1	1
RTN4	0	1	1	1
SON	1	1	1	1
SRP14	1	1	1	1
TNPO1	1	1	1	1
AHNAK	0	1	0	1
ANXA2	0	1	0	1
ARF1	1	0	0	1
CTNNA1	0	1	0	1
EIF5A	1	0	0	1
ENO1	0	1	0	1
FASN	1	1	0	1
GANAB	0	1	0	1
H1FX	1	0	0	1
HNRNPC	1	1	0	1
HNRNPL	1	1	0	1
HSP90AB1	1	1	0	1
JUN	1	0	0	1
KHSRP	1	1	0	1
MAP4	1	1	0	1
MEX3C	1	1	0	1
MYH9	1	0	0	1
NAP1L4	0	1	0	1
PLEC	0	1	0	1
PRRC2B	1	1	0	1
QKI	1	0	0	1
RDX	0	1	0	1
SERPINH1	0	1	0	1
SNRNP200	1	1	0	1
SRPR	0	1	0	1
ZMAT3	0	1	0	1
ARCN1	0	1	1	0
ATP5A1	0	1	1	0
BCLAF1	1	1	1	0
CAPRIN1	1	1	1	0
CCDC47	0	1	1	0
CDKN2A	1	0	1	0
CCT4	0	1	1	0
CHERP	1	1	1	0
CNBP	1	1	1	0
CSDE1	1	1	1	0
CPSF6	1	1	1	0

DDX1	1	1	1	0
DDX21	1	1	1	0
DDX3X	1	1	1	0
DEK	1	1	1	0
DHX9	1	1	1	0
DKC1	0	1	1	0
EIF1AX	1	1	1	0
EIF2S1	1	1	1	0
EIF3E	1	0	1	0
ELAVL1	1	1	1	0
EIF4B	1	1	1	0
ERH	1	0	1	0
FYTTD1	1	0	1	0
FUBP1	1	1	1	0
GDI2	1	0	1	0
G3BP1	1	1	1	0
H1F0	1	1	1	0
HDGF	0	1	1	0
HLTF	0	1	1	0
HNRNPA0	1	1	1	0
HNRNPA2B1	1	1	1	0
HNRNPA3	1	1	1	0
HMGB2	1	1	1	0
HNRNPA1	1	1	1	0
HNRNPD	1	1	1	0
HNRNPH3	1	1	1	0
HNRNPF	1	1	1	0
HNRNPH1	1	1	1	0
HNRNPH2	1	1	1	0
HNRNPR	1	1	1	0
HSPE1	0	1	1	0
HSPA9	1	1	1	0
IGF2BP1	1	1	1	0
IGF2BP3	1	1	1	0
KHDRBS1	1	1	1	0
LARP1	1	1	1	0
LBR	1	0	1	0
LARP4	1	1	1	0
LRPPRC	1	1	1	0
LIN28B	1	0	1	0
MAPRE1	1	0	1	0
MATR3	1	1	1	0
MEX3A	1	0	1	0
MRPL42	0	1	1	0
MRPS28	1	1	1	0
MSI2	1	1	1	0
MYEF2	1	0	1	0
NAP1L1	1	0	1	0
NCBP2	1	1	1	0
NOLC1	1	1	1	0
NPM1	1	1	1	0
NUDT21	1	1	1	0
NUSAP1	0	1	1	0
PABPC1	1	1	1	0
PCBP1	1	1	1	0
PCBP2	1	1	1	0
PEG10	1	1	1	0
PNN	1	1	1	0
PPP1CC	1	0	1	0

PRKDC	1	0	1	0
PTBP2	1	1	1	0
RAN	1	1	1	0
RBFOX2	1	1	1	0
RBM12	0	1	1	0
RBM14	1	1	1	0
RBMX	1	1	1	0
RPL15	1	1	1	0
RPL23A	1	1	1	0
RPL27A	1	0	1	0
RPS12	1	1	1	0
RPS23	1	0	1	0
SF3B1	1	1	1	0
SFPQ	1	1	1	0
SLBP	0	1	1	0
SMC1A	1	0	1	0
SMG1	1	0	1	0
SRSF1	1	1	1	0
SRSF2	0	1	1	0
SRSF6	0	1	1	0
STRAP	1	1	1	0
SUMO2	1	0	1	0
SYNCRIP	1	1	1	0
TFAM	1	1	1	0
TFRC	0	1	1	0
U2AF1	1	1	1	0
UPF3B	1	1	1	0
XRCC5	1	1	1	0
YWHAE	0	1	1	0
YWHAZ	0	1	1	0
ZNF638	1	1	1	0

Supplementary Table 10: SU_MB002 and NSC201cb MSI1 eCLIP bound gene annotated as LncRNA

Cell line	Log_2FC	Strand	Ensembl ID	Gene ID	Binding region
SU_MB002	3.676006578	-	ENSG00000260032.1	LINC00657	noncoding_exon
	3.825131127	-	ENSG00000245910.4	SNHG6	noncoding_exon
	3.904195725	+	ENSG00000233137.2	RP11-220I1.1	noncoding_exon
	3.556087385	+	ENSG00000251562.3	MALAT1	noncoding_exon
	3.831356997	-	ENSG00000205663.5	RP11-706O15.5	distnoncoding_intron500
	4.997710964	+	ENSG00000247556.2	OIP5-AS1	noncoding_exon
	3.429350646	-	ENSG00000260032.1	LINC00657	noncoding_exon
NSC201cb	4.733314744	+	ENSG00000260054.1	RP11-611L7.1	noncoding_exon
	3.305182483	-	ENSG00000188825.9	LINC00910	noncoding_exon
	3.688681075	-	ENSG00000176728.3	TTTY14	distnoncoding_intron500
	4.166164198	-	ENSG00000260032.1	LINC00657	noncoding_exon
	4.810191681	+	ENSG00000225783.2	MIAT	noncoding_exon

Supplementary Table 11: Top 50 polysome-associated transcripts differentially abundant (FDR<0.01) in control vs sh*MSI1* inhibited samples identified and quantified

ENSEMBL ID	GENEID	Fold change	*p*-value
ENSG00000179743.2	MST1P2	1.499847961	0.00386915
ENSG00000131652.9	THOC6	1.410367239	0.001484389
ENSG00000185269.7	NOTUM	1.389393455	0.006047877
ENSG00000177697.13	CD151	1.354322202	0.005839317
ENSG00000165644.6	COMTD1	1.348425321	0.000931943
ENSG00000020129.11	NCDN	1.335692878	0.000964438
ENSG00000119383.15	PTPA	1.334193996	0.002659208
ENSG00000156521.9	TYSND1	1.315562872	0.005364365
ENSG00000092621.7	PHGDH	1.312024166	0.004493449
ENSG00000136270.9	TBRG4	1.284589739	0.006032564
ENSG00000173456.4	RNF26	1.267032772	0.004698971
ENSG00000168476.7	REEP4	1.265832607	0.00125763
ENSG00000095059.11	DHPS	1.258219552	0.002755163
ENSG00000148308.13	GTF3C5	1.2514387	0.002497531
ENSG00000198937.8	CCDC167	1.236401661	0.00602013
ENSG00000186312.6	CA5BP1	1.234390476	0.000905036
ENSG00000173898.7	SPTBN2	1.22416195	0.004929378
ENSG00000068120.10	COASY	1.21793611	0.002618061
ENSG00000113758.9	DBN1	1.21116594	0.005939095
ENSG00000149930.13	TAOK2	1.201121449	0.003119549
ENSG00000204356.7	NELFE	1.192791602	0.003632649
ENSG00000114650.14	SCAP	1.189517546	0.003931273
ENSG00000185252.13	ZNF74	1.189003602	0.005092699
ENSG00000163156.7	SCNM1	1.159669524	0.005415498
ENSG00000114554.7	PLXNA1	1.147781406	0.002047065
ENSG00000089123.11	TASP1	0.779690031	0.005660311
ENSG00000100568.6	VTI1B	0.762955768	0.006190014
ENSG00000153879.4	CEBPG	0.730222032	0.004408336
ENSG00000119185.8	ITGB1BP1	0.71626293	0.006700319
ENSG00000224568.1	LINC01886	0.694487509	0.005797613
ENSG00000147316.8	MCPH1	0.664667565	0.006732803
ENSG00000214413.3	BBIP1	0.657047158	0.00600773
ENSG00000143933.12	CALM2	0.625443389	0.002395107
ENSG00000181163.9	NPM1	0.608587743	0.004319377
ENSG00000225648.1	SBDSP1	0.60467543	0.002911525
ENSG00000117906.9	RCN2	0.595801594	0.004441108
ENSG00000249353.2	NPM1P27	0.585360198	0.002074105
ENSG00000134748.12	PRPF38A	0.578071959	0.004686736
ENSG00000197062.7	ZSCAN26	0.554782057	0.001300728
ENSG00000111726.8	CMAS	0.54407434	0.00247979
ENSG00000198315.6	ZKSCAN8	0.5282526	0.000881507
ENSG00000164291.12	ARSK	0.508705373	0.000178418
ENSG00000129515.14	SNX6	0.425462713	0.003220632
ENSG00000181315.6	ZNF322	0.423300997	0.006740281
ENSG00000168803.10	ADAL	0.382974686	0.001757012
ENSG00000215630.5	GUSBP9	0.35680826	0.005908574
ENSG00000146918.15	NCAPG2	0.300753336	0.006605429
ENSG00000132294.9	EFR3A	0.293522273	0.006711303
ENSG00000065243.14	PKN2	0.280822939	0.001273055
ENSG00000126653.11	NSRP1	0.261720751	0.005374746

Supplementary Table 12: Top 50 proteins differentially abundant (FDR<0.01) in control vs sh*MSI1* inhibited samples identified and quantified

Uniprot ID	GENE ID	Fold change	Significant B *p*-value (FDR<0.01)
Q5BKX6	SLC45A4	4.846941677	5.80E-92
P78324	SIRPA	3.347755699	2.54E-28
P30613	PKLR	4.671123379	2.77E-25
Q07617	SPAG1	2.329673661	8.02E-22
P68431	HIST1H3A	2.179437941	1.69E-17
Q13886	KLF9	2.635971199	1.96E-17
O75494	SRSF10	2.906584799	4.22E-17
P62805	HIST1H4A	2.131392532	1.73E-15
P80294	MT1H	2.796504093	2.48E-15
P04004	VTN	2.341765012	1.50E-14
Q96HD1	CRELD1	2.438947711	2.44E-14
P13640	MT1G	3.513815422	1.66E-12
Q86VI3	IQGAP3	2.535168787	2.06E-12
P20336	RAB3A	2.103624306	1.09E-11
Q9UL25	RAB21	2.006665815	1.91E-11
P01308	INS	1.898978317	8.80E-11
Q96ME7	ZNF512	2.208340497	5.00E-10
Q96AQ6	PBXIP1	1.928185476	6.71E-10
P46013	MKI67	1.684183221	6.94E-10
P04733	MT1F	2.174388867	1.47E-09
Q6ZN17	LIN28B	1.88662415	3.93E-09
Q96FQ6	S100A16	2.340069495	4.23E-09
Q96BY9	SARAF	2.118770666	1.03E-08
Q9Y5S1	TRPV2 VRL	2.061482458	1.21E-08
Q58FF6	HSP90AB4P	2.0013563	1.39E-08
P27824	CANX	1.626007741	1.62E-08
Q5SRI9	MANEA	2.232466419	2.06E-08
Q8NBN3	TMEM87A	1.832099759	3.55E-08
P09917	ALOX5	2.933063832	7.04E-08
Q9P2F8	SIPA1L2	2.001874046	8.18E-08
P16401	HIST1H1B	1.742238787	8.64E-08
Q9UIG0	BAZ1B	1.752624689	9.15E-08
Q5SSJ5	HP1BP3	1.854996629	1.30E-07
P48443	RXRG	2.030938152	1.48E-07
Q9BZB8	CPEB	2.871469982	1.86E-07
P02787	TF PRO1400	1.575325761	2.04E-07
Q9BXD5	NPL	2.179095834	2.53E-07
Q15714	TSC22D1	2.006106404	3.02E-07
Q9BVS4	RIOK2	1.820320742	5.43E-07
Q8IXH6	TP53INP2	2.093728856	7.24E-07
P11021	HSPA5	1.546775346	7.77E-07
Q14119	VEZF1	1.874691939	8.41E-07
P11387	TOP1	1.682218394	1.19E-06
Q8WWK9	CKAP2	1.689100083	1.39E-06
Q9BT88	SYT11	2.03813364	1.90E-06
Q9UN86	G3BP2	2.08688852	2.10E-06
Q16666	IFI16	1.661222959	2.46E-06
Q2TB10	ZNF800	2.00857166	3.80E-06
Q12767	TMEM94	0.286376723	6.28E-06
P35243	RCVRN	1.62841598	1.09681E-

Supplementary Table 13: Top 50 genes after RRA analysis that were significantly altered by MSI1 perturbation

Gene	Score	eCLIP logFC	eCLIP -Log1₀IDR	RNA logFC	RNA Adj p-val	Polysome FC	Polysome p-value	Protein FC	Protein SigB
TMEM33	2.004E-05	4.0225609	3.7882789	0.5468676	0.00638716	0.7162411	0.01064035	1.46882199	0.0040081
FBRSL1	2.568E-05	NA	NA	-0.46415	0.00813388	1.1733124	0.04814604	0.64840728	0.0196126
SYP	3.819E-05	NA	NA	-0.632401	0.00020916	1.2562499	0.00906169	1.38817309	0.0513009
CHN1	3.943E-05	NA	NA	-0.95098	2.1054E-05	0.5890674	0.07481982	0.78165816	0.0996516
TNC	4.61E-05	NA	NA	-2.310781	1.4516E-10	NA	NA	NA	NA
SPAG1	8.485E-05	NA	NA	-2.016176	1.1367E-08	NA	NA	2.32967366	1E-16
PLXNA1	9.825E-05	NA	NA	-0.592285	0.00331693	1.1477814	0.00204706	0.77118867	0.0858802
SFXN3	0.000103	3.1154406	4.3267475	0.7040949	8.8303E-05	1.2905154	0.09885239	1.43528779	0.0017224
PTMS	0.0001061	NA	NA	NA	NA	1.3146324	0.02585126	0.59065837	0.0094868
RAB21	0.0001069	4.0824746	3.9639175	1.5577536	1.7156E-06	NA	NA	2.00666582	1E-16
PBXIP1	0.0001086	NA	NA	-1.453728	2.7066E-08	NA	NA	1.92818548	6.706E-10
MKI67	0.0001137	NA	NA	NA	NA	NA	NA	1.68418322	6.936E-10
HIPK1	0.000166	3.9740581	4.147716	0.6070359	0.00528962	0.516091	0.08046359	0.6298787	0.0270068
RXRG	0.0001775	NA	NA	2.6928533	7.0676E-10	NA	NA	2.03093815	1.477E-07
ARL6IP1	0.0002188	7.2592292	52.577568	0.4651254	0.03900659	0.6377301	0.08401567	1.36818006	0.088115
ARL6IP5	0.0002387	NA	NA	0.8232957	8.0931E-05	NA	NA	1.65555527	0.0006904
EEF1A2	0.0003136	NA	NA	3.2270503	2.6732E-08	1.4016697	0.04478539	1.54014751	0.0008971
APOOL	0.0003381	NA	NA	NA	NA	0.5165279	0.04225632	1.2842832	0.0494049
ABCC4	0.0003657	NA	NA	1.2216924	3.8041E-07	NA	NA	NA	NA
ELAVL2	0.0003791	NA	NA	-0.968007	0.00040565	NA	NA	1.33890715	0.0147838
TMEM245	0.0004211	NA	NA	1.2978042	6.8562E-06	NA	NA	1.53494086	0.0093587
HSPA14	0.0004552	NA	NA	NA	NA	0.7035168	0.02935022	1.31074982	0.058125
SOAT1	0.0004595	3.7305119	3.4256777	2.0358554	7.814E-09	NA	NA	1.39088547	0.055
ST6GALNAC5	0.0004849	NA	NA	-1.128316	1.5458E-07	NA	NA	1.85609687	0.0001242
AGPAT5	0.0005025	NA	NA	NA	NA	0.5991401	0.0105837	1.27034819	0.0619576
TMSB15B	0.000553	NA	NA	-0.989033	0.0013512	0.7196913	0.03001048	0.42924419	0.0003156
GPR180	0.0005731	NA	NA	NA	NA	0.6002032	0.05217023	1.52729707	0.0040838
AKT3	0.0005969	NA	NA	1.3421005	1.055E-06	NA	NA	NA	NA
PRDX3	0.000678	NA	NA	1.2497427	6.933E-07	NA	NA	1.46655594	0.0007611
MAOA	0.0007076	NA	NA	1.2562767	1.2765E-06	NA	NA	1.51241108	0.0002226
RAB12	0.0007205	NA	NA	NA	NA	NA	NA	1.9184592	2.785E-05
DBN1	0.0007636	NA	NA	-0.788846	5.0799E-06	1.2111659	0.0059391	NA	NA
ARSK	0.0007926	NA	NA	NA	NA	0.5087054	0.00017842	NA	NA
ID1	0.0007926	NA	NA	5.9635835	5.7438E-12	NA	NA	NA	NA
INS-IGF2	0.0007926	NA	NA	NA	NA	NA	NA	1.89897832	1E-16
SEC23B	0.0007926	NA	NA	NA	NA	NA	NA	NA	NA
TRPV2	0.000843	NA	NA	0.8698743	0.00249904	1.2963345	0.03910161	2.06148246	1.209E-08
SOCS2	0.0009171	NA	NA	-1.395216	4.9622E-05	0.6629625	0.06010278	0.70268279	0.0457516
SBDS	0.0009311	NA	NA	NA	NA	0.5686119	0.05826656	1.23789041	0.0877462
TNKS	0.0010628	NA	NA	NA	NA	0.446954	0.04860425	1.39652714	0.050171
CHRNA9	0.0010897	NA	NA	0.8420491	2.2639E-05	0.5649382	0.02905636	NA	NA
ARHGEF2	0.0011979	NA	NA	-0.607666	0.00400676	1.2618586	0.05723325	1.23206083	0.0958593
STMN2	0.0012271	NA	NA	-0.485033	0.004192	NA	NA	0.69653094	0.0587594
PAIP2	0.0013844	NA	NA	NA	NA	0.6428044	0.06724992	0.73141933	0.0476683
SLC30A1	0.0015448	NA	NA	0.7577721	0.00310099	0.5839775	0.06943776	1.41837995	0.0373964
ELAVL3	0.0015672	NA	NA	-1.615813	1.5458E-07	NA	NA	NA	NA
ERCC1	0.0015851	4.4087661	4.7242873	NA	NA	NA	NA	NA	NA
INS	0.0015851	NA	NA	NA	NA	NA	NA	1.89897832	1E-16
SAMD11	0.0015851	NA	NA	6.0114594	5.7438E-12	NA	NA	NA	NA

Supplementary Table 14: Reactome functional interaction network MYC association with top RRA genes

Gene1	Gene2	Annotation	Direction	Score
CDK6	MYC	Gene expression interaction: Relation of transcription factor and target gene product	<-	1
CKS1B	MYC	Gene expression interaction: Relation of transcription factor and target gene product	<-	1
EPN1	MYC	expression regulated by	<-	1
HIPK1	MYC	expression regulated by	<-	1
JUN	MYC	expression regulates	->	1
LBH	MYC	expression regulated by	<-	1
MMP2	MYC	Gene expression interaction: Relation of transcription factor and target gene product	<-	1
MYC	NFIC	expression regulated by	<-	1
MYC	NFYA	activated by; complex; input	<-	1
MYC	NME1	expression regulates	->	1
MYC	PIK3R1	activated by	<-	1
MYC	STAT5A	Gene expression interaction: Relation of transcription factor and target gene product	<-	1
MYC	STAT5B	Gene expression interaction: Relation of transcription factor and target gene product	<-	1
MYC	THRB	Gene expression interaction: Relation of transcription factor and target gene product	<-	1

Supplementary Table 15: HIPK1 and MYC shared annotated biological pathways

GO and REACTOME ID	Biological process
GO:0002376	Immune system process
GO:0002520	Immune system development
GO:0006464	Cellular protein modification process
GO:0006468	Protein phosphorylation
GO:0006793	Phosphorus metabolic process
GO:0006796	Phosphate-containing compound metabolic process
GO:0006807	Nitrogen compound metabolic process
GO:0006915	Apoptotic process
GO:0006950	Response to stress
GO:0006974	Cellular response to DNA damage stimulus
GO:0007154	Cell communication
GO:0007165	Signal transduction
GO:0007166	Cell surface receptor signaling pathway
GO:0007275	Multicellular organism development
GO:0007399	Nervous system development
GO:0008150	Biological process
GO:0008152	Metabolic process
GO:0008219	Cell death
GO:0008283	Cell population proliferation
GO:0008284	Positive regulation of cell population proliferation
GO:0009653	Anatomical structure morphogenesis
GO:0009790	Embryo development
GO:0009887	Animal organ morphogenesis
GO:0009966	Regulation of signal transduction
GO:0009987	Cellular process
GO:0010033	Response to organic substance
GO:0010646	Regulation of cell communication
GO:0012501	Programmed cell death
GO:0016043	Cellular component organization
GO:0016310	Phosphorylation
GO:0019221	Cytokine-mediated signaling pathway
GO:0019538	Protein metabolic process

GO:0022008	Neurogenesis
GO:0022603	Regulation of anatomical structure morphogenesis
GO:0022607	Cellular component assembly
GO:0023051	Regulation of signaling
GO:0023052	Signaling
GO:0030097	Hemopoiesis
GO:0030154	Cell differentiation
GO:0032501	Multicellular organismal process
GO:0032502	Developmental process
GO:0033554	Cellular response to stress
GO:0034097	Response to cytokine
GO:0035239	Tube morphogenesis
GO:0035295	Tube development
GO:0035556	Intracellular signal transduction
GO:0036211	Protein modification process
GO:0042127	Regulation of cell population proliferation
GO:0042221	Response to chemical
GO:0043170	Macromolecule metabolic process
GO:0043412	Macromolecule modification
GO:0044085	Cellular component biogenesis
GO:0044237	Cellular metabolic process
GO:0044238	Primary metabolic process
GO:0044260	Cellular macromolecule metabolic process
GO:0044267	Cellular protein metabolic process
GO:0048513	Animal organ development
GO:0048518	Positive regulation of biological process
GO:0048522	Positive regulation of cellular process
GO:0048534	Hematopoietic or lymphoid organ development
GO:0048583	Regulation of response to stimulus
GO:0048699	Generation of neurons
GO:0048731	System development
GO:0048856	Anatomical structure development
GO:0048869	Cellular developmental process
GO:0050789	Regulation of biological process
GO:0050793	Regulation of developmental process
GO:0050794	Regulation of cellular process
GO:0050896	Response to stimulus
GO:0051094	Positive regulation of developmental process
GO:0051239	Regulation of multicellular organismal process
GO:0051240	Positive regulation of multicellular organismal process
GO:0051716	Cellular response to stimulus
GO:0065007	Biological regulation
GO:0070887	Cellular response to chemical stimulus
GO:0071310	Cellular response to organic substance
GO:0071345	Cellular response to cytokine stimulus
GO:0071704	Organic substance metabolic process
GO:0071840	Cellular component organization or biogenesis
GO:1901564	Organonitrogen compound metabolic process
GO:1902531	Regulation of intracellular signal transduction
GO:2000026	Regulation of multicellular organismal development
GO:0005575	Cellular component
GO:0005622	Intracellular
GO:0005623	Cell
GO:0005634	Nucleus
GO:0005654	Nucleoplasm
GO:0005737	Cytoplasm
GO:0031974	Membrane-enclosed lumen
GO:0031981	Nuclear lumen
GO:0043226	Organelle
GO:0043227	Membrane-bounded organelle
GO:0043228	Non-membrane-bounded organelle
GO:0043229	Intracellular organelle
GO:0043231	Intracellular membrane-bounded organelle
GO:0043232	Intracellular non-membrane-bounded organelle

GO:0043233	Organelle lumen
GO:0044422	Organelle part
GO:0044424	Intracellular part
GO:0044428	Nuclear part
GO:0044444	Cytoplasmic part
GO:0044446	Intracellular organelle part
GO:0044464	Cell part
GO:0070013	Intracellular organelle lumen
GO:0003674	Molecular function
GO:0003676	Nucleic acid binding
GO:0003677	DNA binding
GO:0005488	Binding
GO:0005515	Protein binding
GO:0097159	Organic cyclic compound binding
GO:1901363	Heterocyclic compound binding
REAC:R-HSA-212436	Generic Transcription Pathway
REAC:R-HSA-73857	RNA Polymerase II Transcription
REAC:R-HSA-74160	Gene expression (Transcription)

Supplementary Table 16: SU_MB002 and NSC201cb MSI1 eCLIP bound to stop codons of their target genes

Cell line	Gene name	Chromosome	Start	Stop	Strand	-log$_{10}p$	Log2FC
SU_MB002	COX7C	chr5	85916483	85916573	+	14.153699	5.56689
	GLS	chr2	191797526	191797604	+	3.972774	3.988932
	H2AFV	chr7	44869663	44869768	-	3.026451	3.544489
	ATP2A2	chr12	110785207	110785281	+	6.053567	4.712133
	CCNB1IP1	chr14	20779691	20779784	-	5.31186	4.158675
	SLC6A15	chr12	85255583	85255670	-	3.5866	3.709468
	HSPE1	chr2	198367931	198368052	+	12.626707	4.406231
	PPAT	chr4	57261431	57261528	-	18.007242	5.787671
	SRP14	chr15	40328535	40328621	-	4.154761	3.164025
	ODC1	chr2	10580842	10580858	-	3.368256	3.78028
	HNRNPA3	chr2	178084014	178084042	+	4.098051	3.287652
	SOD1	chr21	33040880	33040992	+	6.68351	4.597048
	RBBP4	chr1	33146119	33146223	+	4.487784	3.273442
	DDAH2	chr6	31694847	31694994	-	4.098051	3.269155
NSC201cb	RGMA	chr15	93588204	93588231	-	19.655077	3.301307
	MAP4	chr3	47894223	47894328	-	23.22715	3.83863
	DDR1	chr6	30867812	30867865	+	38.976167	6.836803
	CD59	chr11	33731582	33731685	-	10.843148	3.217104
	B2M	chr15	45008526	45008542	+	6.550825	3.729693
	LUZP6	chr7	135611935	135612059	-	8.381997	5.566891
	SERF2	chr15	44086144	44086198	+	5.076865	3.421619
	APP	chr21	27253915	27253993	-	27.235368	3.710974
	SQSTM1	chr5	179263638	179263737	+	34.090171	4.730303
	SEPT11	chr4	77957888	77957959	+	9.953817	4.391434
	ATP2A2	chr12	110785158	110785240	+	48.183955	5.745688
	ANXA5	chr4	122589547	122589643	-	17.45715	4.261117
	CTSB	chr8	11702589	11702634	-	8.902724	4.047896
	SEPT11	chr4	77955658	77955702	+	5.135449	4.65041
	PTPRZ1	chr7	121701247	121701309	+	35.592266	5.826827
