# Supplementary material for: Characterization of an RNA binding protein interactome reveals a context-specific post-transcriptional landscape of MYC-amplified medulloblastoma
Source: Nat Commun. 2022 Dec 6;13:7506. doi: 10.1038/s41467-022-35118-3 (PMC9726987; doi:10.1038/s41467-022-35118-3)
Supplement: Supplementary file 3 — Reporting Summary [file 41467_2022_35118_MOESM3_ESM.pdf]

nature research

Corresponding author(s): Dr. Robert J Wechsler-Reya*
Dr. Gene W Yeo*
Dr. Sheila K Singh*

Last updated by author(s): Nov 1, 2022

Reporting Summary

Nature Research wishes to improve the reproducibility of the work that we publish. This form provides structure for consistency and transparency in reporting. For further information on Nature Research policies, see our Editorial Policies and the Editorial Policy Checklist.

Statistics

For all statistical analyses, confirm that the following items are present in the figure legend, table legend, main text, or Methods section.

n/a	Confirmed	
☐	☒	The exact sample size (*n*) for each experimental group/condition, given as a discrete number and unit of measurement
☐	☒	A statement on whether measurements were taken from distinct samples or whether the same sample was measured repeatedly
☐	☒	The statistical test(s) used AND whether they are one- or two-sided<br>*Only common tests should be described solely by name; describe more complex techniques in the Methods section.*
☐	☒	A description of all covariates tested
☐	☒	A description of any assumptions or corrections, such as tests of normality and adjustment for multiple comparisons
☐	☒	A full description of the statistical parameters including central tendency (e.g. means) or other basic estimates (e.g. regression coefficient) AND variation (e.g. standard deviation) or associated estimates of uncertainty (e.g. confidence intervals)
☐	☒	For null hypothesis testing, the test statistic (e.g. *F*, *t*, *r*) with confidence intervals, effect sizes, degrees of freedom and *P* value noted<br>*Give P values as exact values whenever suitable.*
☐	☒	For Bayesian analysis, information on the choice of priors and Markov chain Monte Carlo settings
☒	☐	For hierarchical and complex designs, identification of the appropriate level for tests and full reporting of outcomes
☐	☒	Estimates of effect sizes (e.g. Cohen's *d*, Pearson's *r*), indicating how they were calculated

Our web collection on statistics for biologists contains articles on many of the points above.

Software and code

Policy information about availability of computer code

Data collection

eCLIP and RNA-seq data was collected using Illumina Hiseq 2000 platform (Illumina, San Diego, CA, USA) at McMaster University, Polysome profiling-seq data was collected using, mass spectrometric (LC-MS) data was collected using a Thermo Fisher UltiMateTM 3000 RSLCNano UPLC system that ran a 3hr gradient at 70nL/min, coupled to a Thermo QExactive HF quadrupole-Orbitrap mass spectrometer. Flow cytometry data was collected using MoFlo XDP cell sorter (Beckman Coulter).

Data analysis

The pipeline used to process the eCLIP data is available and described in the can Nostrand et al 2016 (http://yeolab.github.io/papers/2016/nmeth_eric_2016.pdf). eCLIP reads were processed and QC was performed according to the ENCODE data processing protocol for eCLIP reads as previously described 24. First, reads were demultiplexed according to their inline barcodes (MB002_Msi1: A01, B06; NSC201cb_Msi1: X2A, X2B) using a custom script, which also modifies each read name to include the read's unique molecular identifier (UMI) (demux.py). Next, reads were trimmed using cutadapt (v1.14) and filtered of any read mapped to RepBase (v18.05) sequences using STAR (v2.4.0j). Surviving reads were then mapped, again with STAR, to hg19 assembly to obtain genome alignments. PCR duplicate removal was then performed with a custom script based on UMI sequences placed inside each read name (barcodecollapsepe.py). De-duplicated mapped BAM files from each barcode were then combined (samtools merge v1.6), forming a single BAM file for each single IP and size-matched INPUT dataset. Read2 for each IP merged BAM file were used to call enriched peak clusters with Clipper (v1.2.1). These clusters were then normalized against size-matched INPUT reads and neighboring/overlapping clusters merged. Regions passing a -log10(p) significance of at least 3 and a log2(fold change) cutoff of 3 were deemed as significantly Msi1-bound for each replicate. To obtain reproducible regions between two replicates, we used the modified IDR pipeline as previously described 94. Using the outputs from the processing pipeline, input normalized peaks were ranked according to information content (pi*log2(pi/qi). These ranked peaks were passed to IDR (v2.0.2) to determine regions of reproducibility. Full definitions for each tool and workflow can be found at: https://github.com/YeoLab/merge_peaks. The demultiplex script can be found at: https://github.com/YeoLab/eclipdemux. The pipeline definitions and barcode collapse script can be found at: https://github.com/YeoLab/eclip.
For region based fold-enrichment analysis, briefly, mapped reads were counted along all transcripts in Gencode v19 ('comprehensive'). Reads were assigned to all transcripts annotated in Gencode v19. For reads overlapping >1 annotated region, each read was assigned to a single region with the following descending priority order: CDS, 5’UTR, 3’UTR. For each gene, reads were summed up across each region to calculate

final region counts. A minimum of 10 observed reads were required for a gene to be considered in region-based fold-enrichment analyses. Motif analysis was performed using HOMER (v4.9.1) wrapped inside a custom script (analyze_motifs.py found here: https://github.com/YeoLab/clip_analysis_legacy). The methodology was first described by Lovci et al95; briefly peaks were assigned to their corresponding regions of binding (CDS, 3'UTR, 5'UTR, proximal and distal intron +/- 500bp of an exon), then compared against a randomized background (random assignments of peak coordinates across each corresponding region).

The RNA-seq: Filtered reads were mapped to the human genome (hg19) using the STAR short-read aligner (v.2.4.2a) with the following command: STAR --genomeDir /path/to/GRCh37 --readFilesIn <file1.fastq.gz> <file2.fastq.gz> --readFilesCommand zcat --runThreadN 8 --outSAMstrandField intronMotif --outSAMtype BAM SortedByCoordinate --quantMode GeneCounts --sjdbGTFfile /path/to/gtf. The gencode.v19.annotation.gtf from the GENCODE database and the primary assembly of GRCh37 was used. Approximately 92% of the filtered reads mapped uniquely, and the read counts from each sample were merged into a single matrix using R. The merged read count matrix was used to compute differential expression using the Bioconductor package limma (v3.38.3) as follows. First, transcripts were filtered using filterByExpr(min.count = 10, min.total.count = 15) (edgeR, v3.25.3) and normalized using calcNormFactors(method = “TMM”). A counts per million matrix was created from the normalized count matrix. Differential gene expression was conducted using the lmFit function and ranked using treat. Significant genes were identified using an FDR < 0.05 and absolute log2(fold-change) > 1.

The polysome profiling data: each polysome-sequencing sample were trimmed using cutadapt (v1.4.0) of adaptor sequences and mapped to repetitive elements (RepBase v18.04) using the STAR (v2.4.0i). The filtered reads which did not map to repetitive elements were then mapped to the human genome (hg19). Using GENCODE (v19) gene annotations and featureCounts (v.1.5.0) to create read count matrices. Approximately 90% of the filtered reads mapped uniquely. The transcript RPKMs of input and polysome fractions were calculated from the read count matrices. Only genes with mean of reads > 10 and mean of RPKM > 1 were considered. Polysome association was calculated by RPKM ratio of transcript levels in polysomes over input.

The mass spectrometry data: LC-MS data generated was analyzed against a UniProt human protein database (42,173 entries) for protein identification and quantification by MaxQuant software (v.1.6.5) From 2,379,345 MS/MS spectra acquired in all 38 fractions, 136,833 unique peptide groups (with Peptide FDR<0.01) and 8,547 proteins (Protein FDR < 0.01) were identified and quantified 96. The Significant B values were calculated using the PERSEUS (v.1.6.5) software. Significance B value preset with an FDR<0.01 was used to identify proteins that are significantly differentially abundant and used for downstream integrative analysis.

Pathway analysis for the comparison between eCLIP datasets in SU_MB002 and NSC201 cell lines was conducted using g:Profiler (Reimand et al., 2007). Genes were ranked by decreasing fold change. Gene sets from Reactome (v64, released 2018-10-02) and Gene Ontology databases (version Ensembl v93/ Ensembl Genomes v40, released 2018-08-03) were included. Gene sets were limited to between 5 and 500 genes and pathways were filtered for a statistical threshold of p < 0.05.

Data integration of eCLIP, mRNA, polysome-seq and protein datasets was conducted using Robust Rank Aggregation using default parameters58. For eCLIP, the significance threshold was relaxed to include sites up to –log10IDR > 1 and log2FC > 1. For mRNA and protein, the significance thresholds were relaxed to adj p-value < 0.1 and Sig.B < 0.1 to filter the data. Genes were ranked by statistical significance. Pathway analysis was conducted using gProfileR using the parameters described above. Visualization was done in Cytoscape (v.3.6.0). Data visualization was done using Boutroslab.plotting.general (v.5.9.2)101 and ggplot2 (v3.1.0)102. Data for the ribbon plot for the network diagram was extracted from the Reactome Functional Interaction Database (Wu et al., 2010). Data was visualized using the R package circlize (v0.4.5).

The flow cytometry data: was analyzed using Kaluza 2.0 (Beckman Coulter) gating strategy is provided in Supplementary Figure ().

For manuscripts utilizing custom algorithms or software that are central to the research but not yet described in published literature, software must be made available to editors and reviewers. We strongly encourage code deposition in a community repository (e.g. GitHub). See the Nature Research guidelines for submitting code & software for further information.

Data

Policy information about availability of data

All manuscripts must include a data availability statement. This statement should provide the following information, where applicable:

- Accession codes, unique identifiers, or web links for publicly available datasets
- A list of figures that have associated raw data
- A description of any restrictions on data availability

All raw and processed data has been deposited into public databases. For eCLIP (GSE126263), RNA-seq (GSE126337) and polysome profiling-seq (GSE134597) experiments have been deposited into GEO. For mass spectrometric experiments raw data have been deposited in the ProteomeXchange Consortium via Proteomics Identification (PRIDE). The accession number PXD012432. The Molecular Signature Database was used to annotate proteins during the Protein Set Enrichment Analysis of our label-based mass-spectrometry-based quantitative proteomics. LC-MS data generated was analyzed against a UniProt human protein database (42,173 entries) for protein identification and quantification. Enrichment analysis was performed on sets of significant genes/proteins using the EnrichR database. Gene sets from Reactome (v64, released 2018-10-02) and Gene Ontology databases (version Ensembl v93/ Ensembl Genomes v40, released 2018-08-03) were included.

Field-specific reporting

Please select the one below that is the best fit for your research. If you are not sure, read the appropriate sections before making your selection.

☒ Life sciences ☐ Behavioural & social sciences ☐ Ecological, evolutionary & environmental sciences

For a reference copy of the document with all sections, see nature.com/documents/nr-reporting-summary-flat.pdf

Life sciences study design

All studies must disclose on these points even when the disclosure is negative.

Sample size	Figure legends indicate sample sizes for each experiment. No statistical methods to estimate sample size were used. Instead, data from all cancer cases obtained from the cited repositories were analyzed and p-values from statistical tests used to assess statistical significant and appropriateness of sample sizes. No sample size calculation was made as all available cases in the cited repositories were included.
Data exclusions	We excluded from the study MSI1 binding transcripts that were <3FC to maintain a stringent threshold of robust MSI1 binding as per the Yeo laboratory experience.
Replication	The experiments were performed in biological and technical replicates (at least 2 biological replicates in all experiments). Replication was asssessed by analysis of coefficients of variation and t-test and all attempts at replication were successful.
Randomization	Not applicable to study as we accessed the full patient cases of cited cancer repositories, and study did not involve design and/or recruitment of such repositories.
Blinding	Investigators were blind to the identity of the RNA-seq, polysome profiling-seq and mass spectrometric sample processing. Data analysis of the eCLIP, RNA-seq, polysome proflling-seq and proteomics data was blinded to the bioinformatician. Once the data was available, for labeling of figures produced, the identify of the samples were made available by the first author.

Reporting for specific materials, systems and methods

We require information from authors about some types of materials, experimental systems and methods used in many studies. Here, indicate whether each material, system or method listed is relevant to your study. If you are not sure if a list item applies to your research, read the appropriate section before selecting a response.

Materials & experimental systems

n/a	Involved in the study
☐	☒ Antibodies
☐	☒ Eukaryotic cell lines
☒	☐ Palaeontology and archaeology
☐	☒ Animals and other organisms
☐	☒ Human research participants
☒	☐ Clinical data
☒	☐ Dual use research of concern

Methods

n/a	Involved in the study
☒	☐ ChIP-seq
☐	☒ Flow cytometry
☐	☒ MRI-based neuroimaging

Antibodies

Antibodies used

The following antibodies were purchased from various vendors for the study. For mouse samples: MSI1 (rat, 1:200, eBioscience #14H1), alpha-tubulin (rabbit, 1:1000, Cell Signaling #11H10). For human samples: Western blot: β-tubulin (rabbit; 1:50,000; Abcam #ab6046), GAPDH (mouse; 1:2,000; Abcam #ab8245), HDAC1 (rabbit; 1:500; EMD Millipore #06-720), MSI1 (rabbit; 1:2,000; Abcam #ab52865) HIPK1 (1:500; Abcam #ab90103); Immunohistochemistry: MSI1 primary antibody (rabbit, 1:500, Millipore, #AB5977), Nestin (rabbit, 1:15,000, Millipore, #AB5922), and BMI1 (mouse, 1:500, R&D, #MAB33342) ; Flow cytometry: CD133 with an anti-CD133 human clone REA820 (Miltenyi, #130-112-196), Alexa 647 donkey anti-mouse secondary antibody (1:2000 mouse; Thermo #A-31571) and BMI (Miltenyi, #130-106-736); Puromycylation assay: anti-puromycin antibody (1:1000; Millipore; MABE343),

Validation

Antibodies were either raised against human or mouse proteins and validated by the manufacturer.
Specifically from the vendors:

Anti-mouse MSI1 (western): Antibody specificity was demonstrated by detection of differential basal expression of the target across cell lines owing to their inherent genetic constitution. The expression was observed in Neural Stem Cells and IMR-32 and not in Neural Stem Cells differentiated to Astrocytes and MCF 10A using Musashi-1 Monoclonal Antibody (14H1), eBioscience™ (Product # 14-9896-80) in Western Blot.

Anti-mouse alpha-tubulin (Western): α-Tubulin (11H10) Rabbit mAb detects endogenous levels of total α-tubulin protein, and does not cross-react with recombinant β-tubulin. Species Reactivity: Human, Mouse, Rat, Monkey, D. melanogaster, Zebrafish, Bovine, Pig. Species predicted to react based on 100% sequence homology: Dog

Anti-human MSI1 (Western): ab52865 was shown to specifically react with Musashi 1 / Msi1 in wild-type HAP1 cells as signal was lost in MSI1 knockout cells. Wild-type and MSI1 knockout samples were subjected to SDSPAGE. Ab52865 and ab130007 (Mouse anti Vinculin loadingcontrol) were incubated overnight at 4°C at 1/2000 dilution and 1/20000 dilution respectively. Blots were developed with Goat anti-Rabbit IgG H&L (IRDye 800CW) preabsorbed ab216773 and Goat anti-Mouse IgG H&L (IRDye 680RD) preabsorbed ab216776 secondary antibodies at 1/20000 dilution for 1 hour at room temperature before imaging.

Anti-human GAPDH (Western): This GAPDH antibody can be used as a loading control antibody. GAPDH is a 146 kDa tetramer composed of four 30-40 kDa subunits. There is no cross-reaction with GAPDH from yeast. Preliminary data indicates that the GAPDH

antibody- loading control ab8245 recognizes the monomer (36 kDa) and also the dimer forms of GAPDH, but not the tetrameric form of the protein.

Anti-human HIPK1 (Western): Reacts with: Human, Predicted to work with: Mouse, Rat, Rabbit, Horse, Guinea pig, Cow, Cat, Dog Immunogen Synthetic peptide derived from a region within residues: LNLSQNQQSS AAPTSQERSS NPAPRRQQAF VAPLSQAPYT FQHGSPLHST, corresponding to internal amino acids 1044-1093 of Human HIPK1 (NP_938009).Use a concentration of 1 µg/ml. Predicted molecular weight: 131 kDa. Good results were obtained when blocked with 5% non-fat dry milk in 0.05% PBS-T.

Anti-human beta-tubulin (Western): This polyclonal antibody detects a single clean band at 50kD representing beta Tubulin. This band is significantly reduced by using peptide blocking. Reacts with: Mouse, Rat, Chicken, Human, Pig, Xenopus laevis, Zebrafish, Chinese hamster. Synthetic peptide corresponding to Human beta Tubulin aa 1-100 conjugated to keyhole limpet haemocyanin.
Anti-CD133 (flow cytometry): Clone REA820 recognizes the epitope 2 of the human CD133 antigen (CD133/2). CD133 is a marker that is frequently found on multipotent progenitor cells, including immature hematopoietic stem and progenitor cells, in human fetal liver, bone marrow, cord blood, and peripheral blood. CD133 has also been found to be expressed on circulating endothelial progenitor cells, tissue-specific stem cells, cancer stem cells from tumor tissues, as well as ES and iPS cell–derived cells.Clone REA820 displays negligible binding to Fc receptors.

Anti-BMI1 (flow cytometry): Clone REA438 recognizes the human B lymphoma Mo-MLV insertion region 1 homolog (BMI-1) antigen, which is also known as polycomb group RING finger protein 4 (PCGF4). BMI-1 is a member of the polycomb group of transcription repressors that was initially identified as an oncogene cooperating with c-myc in a murine model of lymphoma. Both, hematopoietic stem cells (HSCs) and neuronal stem cells, express high levels of BMI-1. It has been shown that BMI-1 is necessary for efficient self-renewing cell divisions of adult HSCs as well as adult peripheral and central nervous system neural stem cells, but that it is less critical for the generation of differentiated progeny. BMI-1 causes neoplastic proliferation when overexpressed in lymphocytes. Clone REA438 displays negligible binding to Fc receptors.

Anti-MSI1 (immunohistochemistry): Polyclonal Antibody. Routinely evaluated by Western Blot on Human Placenta lysates. Western Blot Analysis: 1:1000 dilution of this lot detected Musashi-1 on 10 µg of Human placenta lysates. Synthetic peptide amino acids 5-21 Musashi. Specificity: recognizes Musashi-1. Species reactivity: Human, mouse, rat.

Alexa 647 donkey Anti-mouse secondary antibody (flow cytometry) Flow cytometry analysis of Pax6 on human neural stem cells derived from PD-3 iPSCs using Gibco® PSC Neural Induction Medium (Product # A1647801). Cells were fixed, permeabilized, and then stained with a Pax6 polyclonal antibody (Product # 42-6600) at a 1:100 dilution and a Nestin mouse monoclonal antibody (Product # MA1-110) at a 1:100 dilution. After incubation of the primary antibodies for 1 hour on ice, the cells were stained with Alexafluor® 488-conjugated goat anti-rabbit IgG secondary antibody (Product # A-11034) and Alexafluor® 647-conjugated donkey anti-mouse IgG secondary antibody (Product # A-31571) at a dilution of 1:500 for 1 hour on ice. Flow cytometry analysis was performed using the Attune® Acoustic Focusing Cytometer (Product # 4469120). A representative 10,000 cells were acquired for each sample.

Anti-BMI1 (immunohistochemistry): Anti-BMI1 monoclonal mouse IgG2A Clone # 384509. Immunogen is E. coli-derived recombinant human BMI-1 Asp96-Gly326. Specificity detects hyman BMI-1 in direct ELISAs. Species reactivity: Human.

Anti-Nestin (immunohistochemistry): Anti-Nestin IgG Polyclonal Antibody is an antibody against Nestin for use in IC, IH, IH(P) & WB. 1:200 dilution of a previous lot was used on formalin fixed, paraffin embedded tissue sections. Human Nestin. The antibody shows no reactivity to vimentin or GFAP. Fusion protein. Species reactivity: Human.

Anti-puromycin (Flow cytometry): Anti-Puromycin monoclonal antibody, clone 12D10, detects puromycin incorporated into protein (isotype IgG2aκ). Immunogen: Puromycin from Streptomyces alboniger. Host: Mouse and reactiivty with all species including humans. Monoclonal antibodies to puromycin may be used with standard immunochemical methods. Immunohistochemistry Analysis: A representative lot detecte Puromycin-incorporated neosynthesized protein in IHC (Goodman, C. A., et al. (2010). FASEB J. 25(3):1028-1039.)

Eukaryotic cell lines

Policy information about cell lines

Cell line source(s)

Primary human pediatric MBs, SU_MB002 and HD-MB03 were kind gifts from Dr. Yoon-Jae Cho (Harvard, MS) and Dr. Till Milde (Heidelberg) respectively. The primary human MB, BT853 is a Wnt MB cell line established from fresh tissue section at surgical resection from a 5-year old female. BT853 was established after informed consent from the family and as approved by the Hamilton Health Sciences/McMaster Health Sciences Research Ethics Board.
The development and culturing of MP tumors were completed in the Dr. Wechsler-Reya laboratory.
HEK293T cells were purchased from ATCC Cat#CRL-11268.

Authentication

Cell lines were authenticated using NanoString technology to identify verify their MB subgrouping as previously described.

Mycoplasma contamination

Cell lines were regularly tested for mycoplasma and cultured in Mycozap during expansion prior to use in experiments and were free from contamination.

Commonly misidentified lines
(See ICLAC register)

No commonly misidentified cell lines were used.

Animals and other organisms

Policy information about studies involving animals; ARRIVE guidelines recommended for reporting animal research

Laboratory animals

For mouse G3 MB MSI1 studies, NSG (NOD-SCID IL2R-gamma null) mice were purchased from Jackson Labs were used as hosts for

Laboratory animals	orthotopic tumor transplantation. WT (C57BL/6J) pups for tumors generation were obtained from the Sanford Burnham Prebys (SBP) Medical Discovery Institute Animal Facility. Mice were maintained in the animal facilities at the Sanford Consortium for Regenerative Medicine (SCRM).<br>For human G3 MB MSI1 NOD-SCID mice were purchased from Jackson labs were used as hosts for orthotopic tumor transplantation. Mice were maintained in the animal facilities at the McMaster University Stem Cell Unit (SCU) within the Animal Facility (CAF). All animals were maintained in a pathogen-free, temperature-controlled, 12h light and dark cycle environment and were fed ad libitum
Wild animals	Not applicable, no wild animals were used in the study
Field-collected samples	Not applicable, no field-collected samples were used in the study
Ethics oversight	All in vivo experiments were performed in accordance to the McMaster University Animal Research Ethics Board (AREB) approved protocols national guidelines and regulations, and with the approval of the animal care and use committees at SBP and at the University of California San Diego (UCSD).

Note that full information on the approval of the study protocol must also be provided in the manuscript.

Human research participants

Policy information about studies involving human research participants

Population characteristics	*Describe the covariate-relevant population characteristics of the human research participants (e.g. age, gender, genotypic information, past and current diagnosis and treatment categories). If you filled out the behavioural & social sciences study design questions and have nothing to add here, write "See above."*
Recruitment	*Describe how participants were recruited. Outline any potential self-selection bias or other biases that may be present and how these are likely to impact results.*
Ethics oversight	*Identify the organization(s) that approved the study protocol.*

Note that full information on the approval of the study protocol must also be provided in the manuscript.

Flow Cytometry

Plots

Confirm that:

☒ The axis labels state the marker and fluorochrome used (e.g. CD4-FITC).

☒ The axis scales are clearly visible. Include numbers along axes only for bottom left plot of group (a 'group' is an analysis of identical markers).

☒ All plots are contour plots with outliers or pseudocolor plots.

☒ A numerical value for number of cells or percentage (with statistics) is provided.

Methodology

Sample preparation	shMsi1-GFP transduced MP cells were dissociated with TryplE and suspended in PBS+0.5M EDTA+1%FBS to achieve a single cell suspension with subsequent filtration to remove clumps.
Instrument	MoFlo XDP cell sorter (Beckman Coulter)
Software	Kaluza 2.0 from Beckman Coulter
Cell population abundance	Not applicable (Samples were not sorted)
Gating strategy	Isotype control was used to set the FSC/SSC gate, as well as the boundary for negative staining of CD133 and BMI1 antibodies. Median fluorescence intensity is demonstrated for both isotypes and CD133 and BMI1 antibodies.<br>For the puromycylation assay, cells not treated with puromycin was used to set the median fluorescence intensity for negative staining to compare to median fluorescence intensity of stained and puromycin treated cells.

☒ Tick this box to confirm that a figure exemplifying the gating strategy is provided in the Supplementary Information.

Magnetic resonance imaging

Experimental design

Design type	In vivo imaging of tumor burden
Design specifications	The mouse MRI imaging was performed on a 7 Tesla vertical wide-bore nuclear magnetic resonance (NMR) system (Bruker WB300) using the 30 mm diameter transmit/receive radiofrequency volume coil insert (MicWB40, Bruker Biospin). The protocol allows for tumor visualization without the typical necessity of injectable gadolinium contrast

agents. The mouse is anesthetized in an induction chamber at 5% isoflurane in pure O2 and subsequently positioned in the imaging bed with continuous anesthesia with 1.5-2.5% isoflurane delivered with pure O2 via nose cone. With a combination of stretches, rotations and translations the images are warped into a common alignment, which allows a direct spatial comparison between an animal and a set of healthy controls. Animals were imaged at multiple time points allowing visual detection of tumors less than 0.5 mm diameter confirmed by histopathology.

Behavioral performance measures: Not applicable.

Acquisition

Imaging type(s): Magnetization transfer(MT)-weighted static images

Field strength: 7 Tesla

Sequence & imaging parameters: Whole-head 3D MRI was acquired at 150 µm isotropic voxel resolution, with a FOV of 25 x 25 x 20 mm, with coronal slice orientation, and head-to-foot readout orientation. Magnetization transfer (MT)-weighted images were acquired with a saturation pulse-prepared spoiled gradient echo sequence (MT-FLASH). A Gaussian-shaped saturation pulse was applied once per repetition time (TR), with pulse width: 12 ms, nominal flip angle: 523º (maximum amplitude 6.8 µT), and offset frequency 2500 Hz. The spoiled gradient echo had a TR: 23 ms (including saturation pulse), TE: 3 ms, and excitation angle: 5º. The scan time for a single 3D image was 8.47 minutes. Eight repetitions were performed and registered in post-processing to counteract effects related to motion or tissue settling for a total imaging time of 1.12 hr.

Area of acquisition: Whole mouse brain

Diffusion MRI ☐ Used ☒ Not used

Preprocessing

Preprocessing software: Preprocessing was performed using the FMRIB Software Library (FSL) v5.0. For each mouse, the eight MT-weighted images underwent linear registration (FSL FLIRT) followed by non-linear registration (FSL FNIRT) to the first image in the set. All eight images were then averaged to produce a single MT-weighted image.

Normalization: For visual comparison, images were registered to a study-specific template based on healthy controls.

Normalization template: An age-matched, study-specific template was made from the average of registered (FSL FNIRT) MT-weighted images of mice without tumours from the same strain.

Noise and artifact removal: Motion artifacts were reduced by registering image data from 8 averages per experiment. B1 receive sensitivity gradients (i.e. the "bias field") were removed using FSL FAST.

Volume censoring: Volume censoring was not necessary with this protocol (anaesthetized mice, acquisition time per image sufficient to suppress inter-image differences due to physiological effects).

Statistical modeling & inference

Model type and settings: Images were used solely for visual determination of tumour engraftment, approximate location, and approximate size, therefore statistical models were not applied.

Effect(s) tested: Not applicable. Images were used for assessment of static images with anatomical contrast.

Specify type of analysis: ☒ Whole brain ☐ ROI-based ☐ Both

Statistic type for inference (See Eklund et al. 2016): Not applicable.

Correction: Not applicable.

Models & analysis

n/a	Involved in the study
☒	☐ Functional and/or effective connectivity
☒	☐ Graph analysis
☒	☐ Multivariate modeling or predictive analysis
